# Vascular mimicry as a facilitator of melanoma brain metastasis

**DOI:** 10.1007/s00018-024-05217-z

**Published:** 2024-04-18

**Authors:** Olivia K. Provance, Victor O. Oria, Thuy T. Tran, Jasmine I. Caulfield, Christopher R. Zito, Adam Aguirre-Ducler, Kurt A. Schalper, Harriet M. Kluger, Lucia B. Jilaveanu

**Affiliations:** 1 Department of Medicine, Section of Medical Oncology, Yale University School of Medicine, 333 Cedar Street, SHM234E, New Haven, CT 06520 USA; 2 Biotech Research and Innovation Centre (BRIC), Faculty of Health and Medical Sciences, University of Copenhagen, 2200 Copenhagen, Denmark; 3 Department of Biology, School of Arts, Sciences, Business, and Education, University of Saint Joseph, West Hartford, CT USA; 4 Department of Pathology, Yale University School of Medicine, New Haven, CT USA

**Keywords:** Melanoma, Brain metastasis, Vascular mimicry, YAP/TAZ

## Abstract

Melanoma has the highest propensity among solid tumors to metastasize to the brain. Melanoma brain metastases (MBM) are a leading cause of death in melanoma and affect 40–60% of patients with late-stage disease. Therefore, uncovering the molecular mechanisms behind MBM is necessary to enhance therapeutic interventions. Vascular mimicry (VM) is a form of neovascularization linked to invasion, increased risk of metastasis, and poor prognosis in many tumor types, but its significance in MBM remains poorly understood. We found that VM density is elevated in MBM compared to paired extracranial specimens and is associated with tumor volume and CNS edema. In addition, our studies indicate a relevant role of YAP and TAZ, two transcriptional co-factors scarcely studied in melanoma, in tumor cell-vasculogenesis and in brain metastasis. We recently demonstrated activation of the Hippo tumor suppressor pathway and increased degradation of its downstream targets YAP and TAZ in a metastasis impaired cell line model. In the current study we establish the utility of anti-YAP/TAZ therapy in mouse models of metastatic melanoma whereby treatment effectively inhibits VM and prolongs survival of mice with MBM. The data presented herein suggest that VM may be an important and targetable mechanism in melanoma and that VM inhibition might be useful for treating MBM, an area of high unmet clinical need, thus having important implications for future treatment regimens for these patients.

**Supplementary Information:**

The online version contains supplementary material available at 10.1007/s00018-024-05217-z.

## Introduction

Approximately 40–60% of patients with advanced melanoma are affected by melanoma brain metastases (MBM) [1]. MBM are one of the main causes of death in patients with advanced disease and median survival for patients with untreated MBM is approximately 3–5 months [1–3]. While great progress been made in the treatment of MBM with the emergence of immune checkpoint inhibitors and BRAF- and MEK—targeting drugs, a significant number of patients of patients still fail to respond initially and many progress on treatment [4–8]. Therefore, novel treatments are necessary to improve patient outcomes in MBM.

Development of melanoma metastases is dependent on the ability of disseminated cancer cells to extravasate the circulatory system followed by colonization and outgrowth at distant sites. Like many cancers, melanoma can remain dormant at distant sites for a long duration prior to outbreak from dormancy, which often results in aggressive, overt, and difficult to control disease [9, 10]. A crucial step for reactivation of dormant lesions and sustained tumor growth is activation of quiescent local vasculature to form new vessels for adequate tumor blood supply [10]. Tumors can display three patterns of neovascularization: endothelium dependent vessels (angiogenesis), tumor-cell-lined vessels, and mosaic vessels (combination of endothelial and tumor cells) [11]. There is compelling evidence that a subpopulation of tumor cells can trans-differentiate into vascular endothelial-like cells and form their own functional, vascular-like open-lumen structures to maintain adequate blood supply [12–15]. This unique process of non-angiogenic vascularization, is known as vascular mimicry (VM) [14, 16–18]. Notably, non-angiogenic neovascularization has been shown to support tumor initiation, tumor growth, metastasis, and was linked to poor prognosis in many cancer types [11, 19–21]. VM occurs in multiple cancer types including melanoma [14], breast cancer [22, 23], ovarian cancer [24], prostate cancer [25], glioblastoma [26], renal cell carcinoma [27], and lung cancer [28]. Studies on VM have been scarce, therefore, the extent of the contribution of tumor cells to blood vessels and to the overall tumor blood flow, as well as the pathophysiological and clinically significant role of VM in cancer remain unclear.

The most prominent pathways regulating VM are thought to include Notch, VE-Cadherin and HIF signaling [12, 13], but YAP/TAZ signaling has emerged as a critical mediator of both angiogenesis and vascular mimicry [21, 29–31]. In our previous study, we show that fibromodulin (FMOD), a secreted proteoglycan, modulates the activation of YAP and TAZ, two transcriptional co-factors scarcely studied in melanoma, which induces aggressive variant cells to form VM, and to develop brain metastases [32]. YAP and TAZ have central roles in developmental vasculogenesis and angiogenesis and regulate key functions of endothelial cells. Endothelial YAP/TAZ are critical for the vascular growth, branching, and regularity of blood vessel network. YAP/TAZ induce angiogenesis in endothelial cells through regulation of multiple downstream transcriptional targets such as DKK2, STAT3, ANG2, CTGF and CYR61 [21]. A link between VEGF pathway and YAP and TAZ was found during development and in tumor vasculature [21, 33]. In tumors, aside from facilitating cell proliferation, immune evasion, EMT, and the cancer stem cell phenotype, YAP and TAZ regulate the emergence of pro-angiogenic cancer cells during tumor angiogenesis and play critical roles in VM [21, 30]. While studies to identify target genes of YAP/TAZ that mediate their activity in VM are lacking in melanoma, in other cancers it was found that YAP/TAZ induce VM by regulating the embryonic stem cell transcription factor SOX2 or by upregulating pro-angiogenic factors such as CYR61 and ANG2 [21]. Molecular analysis also uncovered YAP-dependent regulation of MMP2, VE-cadherin, and SMA expression in VM [21]. While these genes explain some phenotypes induced by YAP overexpression and activity, they do not fully account for all YAP/TAZ functions, thus additional target genes and critical drivers of this important process remain to be found.

Current therapies inhibiting tumor neovascularization target pro-angiogenic growth factors including, but not limited to, vascular endothelial growth factor (VEGF) and their receptors (VEGF-receptors) [34, 35]. Examples include bevacizumab, a human monoclonal antibody against VEGF-A and lenvatinib, an anti-angiogenic tyrosine kinase inhibitor which targets VEGFR1-3, but also has activity against fibroblast growth factor receptor (FGFR1-4), platelet-derived growth factor receptor α (PDGFRα), stem cell factor receptor (KIT), and rearranged during transfection (RET) [36, 37]. However, resistance often develops to long-term treatment [34]. Though, in melanoma, adjuvant bevacizumab did not show survival benefit in a phase 3 clinical trial [38], combining anti-VEGF therapies with immunotherapy appears to be an active regimen in advanced melanoma [39]. Previous studies suggest resistance to anti-angiogenic regimens can occur by autocrine and intracrine VEGF/VEGFR2 and YAP/TAZ signaling [29, 31, 40, 41]. Additional studies show that anti-angiogenic agents have little or no effect on VM and can even induce VM formation [42, 43]. In glioblastoma and renal cell carcinoma, non-angiogenic growth was observed in patients who relapsed after anti-angiogenic treatment and was shown to be mechanistically implicated in developing therapy resistance [44–47]. These findings have considerable therapeutic implications for cancer patients receiving anti-angiogenic-based treatments, further highlighting the need for studies focused on VM as an alternative vascularization mechanism.

As non-angiogenic vasculature might precede tumor formation and is associated with metastatic potential and aggressive disease and/or resistance to current therapies, understanding the mechanisms driving VM may lead to the discovery of novel drug targets and the development of new anti-vasculogenic therapies to either prevent expansion of micrometastatic lesions or inhibit the growth of established metastases. In this study we investigated the clinical relevance of VM in intracranial versus extracranial metastatic melanoma and assessed the effect of two YAP/TAZ inhibitors (verteporfin and CA3) on VM formation. This was studied through integration of unique resources including human tumor specimens of paired cerebral and extracerebral melanoma metastases, short-term patient-derived cell lines and relevant murine melanoma models, and functional in vitro and in vivo methods. Herein, we show that VM is more prominent in MBM compared to extracranial metastases in patient samples. Using metastatic melanoma mouse models, we show that while traditional angiogenesis is inhibited by bevacizumab and lenvatinib, these drugs do not alter VM. Lastly, we provide evidence suggesting that pharmaceutically targeting the YAP/TAZ pathway may decrease VM prolonging survival in melanoma brain metastasis.

## Materials and methods

### Tumor microarray and vascular mimicry quantification

Paired intracerebral and extracerebral tumor tissue was obtained from 37 patients with metastatic melanoma who underwent craniotomy between 1997 and 2014 [48]. A tissue microarray including multiple tumor cores for each patient, was constructed to assess blood vessel density and vasculogenic mimicry (VM) thorough microscopic examination of sections for CD34 immunohistochemical and Periodic acid-Schiff (PAS) histochemical staining as explained previously [32]. A feature of VM is a wall structure negative for CD34 but positive for PAS staining, whereas endothelium-lined blood vessels express both CD34 and PAS. The tissue architecture of CD34 + /PAS + vessels for each tumor informed the identification of CD34-/PAS + vessels. CD34 and PAS were stained on sequential cuts, allowing the overlay of CD34 and PAS-stained tissue images to ensure the identified CD34-/PAS + structures were overlapping with or encircled by tumor cells. To enhance rigor of quantification, two independent investigators performed the quantification at both 10x and 20x magnification representative regions from each tumor were quantified to account for site-specific differences in VM between intracerebral and extracerebral melanoma metastasis. The number of VM for each tumor was determined by taking the average of the three representative regions, and the number for each patient was determined by averaging the final score for all site-specific tumors in the array. Data was analyzed blindly.

### Human melanoma cell lines

Cell lines used in this study are early passage (< 20) cultures derived from tumors of melanoma patients treated at Yale University (New Haven, CT). Cell line characteristics and associated citations, if applicable, are listed in Supplementary Table 1. Tumor was collected with the approval of the Yale University Institutional Review Board and banked by the Yale SPORE facility. As previously discussed, [49] mutation patterns were compared with the tumors from which they were derived for authentication. Cell lines derived from melanoma brain metastases include, YUVENA, YUGANK, YUKRIN, YUMETRO and YUTOPIC. Cell lines derived from melanoma tumors at extracranial sites include YUKOLI, YUMUT, YUKSI, YUCOT, YUGASP, YUGEN8, YUSIK, YUSIV. The Cl.2A cell line is a highly brain metastatic derivative of YUGEN8 [32]. As previously described [32], YUGEN8 was subjected to in vitro and in vivo selection to yield the highly brain metastatic clone, Cl.2A, when delivered systemically through left ventricle injection. All human melanoma cell lines were cultured in OptiMEM media (Invitrogen) supplemented with 10% heat-inactivated FBS (Invitrogen) and 1% antibiotic–antimycotic (penicillin, streptomycin, amphotericin B; Invitrogen) at 37 °C in a humidified atmosphere containing 5% CO_2_.

### Mouse melanoma and endothelial cell lines

YUMM1.7, YUMMER1.7, YUMM1.1, and YUMM1.1Br were kindly provided by Marcus Bosenberg (Yale University). YUMM (Yale University Mouse Melanoma) lines were generated as a syngeneic model to C57BL/6 mice [50]. YUMM1.7 and YUMM1.1 are poorly immunogenic tumors and are previously described in detail [50]. YUMMER1.7, an immunogenic counterpart to YUMM1.7, (YUMM Exposed to Radiation) was generated by irradiating YUMM1.7 and expanding a single cell-derived clone [51]. YUMM1.1Br is a brain metastatic derivative generated from harvesting brain metastases formed by injecting YUMM1.1 into the left ventricle of mice. YUMM lines were cultured in DMEM/F12 1:1 media, 10% FBS, 1% AA, 1% NEAA. B16 (ATCC CAT# CRL-6322) and B16.F10 (ATCC CAT# CRL-6475) were purchased from ATCC. B16 and B16.F10 were cultured in OptiMEM media with 7% Horse Serum, 5% FBS, 1% AA at 37 °C in a humidified atmosphere containing 5% CO_2_. Cell line characteristics and associated citations, if applicable, are listed in Supplementary Table 1**.** MS1 (ATCC CAT# CRL-2279) and bEnd.3 (ATCC CAT#CRL-2299) were purchased from ATCC. MS1 were cultured in DMEM with 5% FBS and bEnd.3 were cultured in DMEM with 10% FBS. b.End5 was a gift from the depositor (Culture Collection.org.uk, Cat. #96091930) to Dr. Rafael Reuten at the University of Copenhagen. The normal endothelial cell derived from a healthy brain of Balb/C mouse was purchased from Cell Biologics (Cat.#BALB-5023). bEnd.5 was cultured in DMEM with 10% FBS and normal endothelial cells were cultured in endothelial cell medium (Cell Biologics, Cat.#M1168-Kit) which includes VEGF, heparin, EGF, ECGS, hydrocortisone, antibiotic–antimycotic and FBS. All endothelial cells were cultured at 37 °C in a humidified atmosphere containing 5% CO_2_.

### Vascular mimicry assay

Vascular mimicry assay was completed as described previously [32]. Briefly, 100 µl chilled Matrigel (Corning or R&D) was added to a chilled 48-well plate and incubated for at least 30 min at 37 °C to allow Matrigel polymerization. 30,000 melanoma cells in 300 µL were seeded dropwise to each well in low serum media apart from the YUMM lines being seeded in full media. In experiments including YAP/TAZ inhibitors, drugs were added to the mixture of cells and media prior to adding dropwise to the well. Vascular mimicry was evaluated by microscopy at 6 h. Multiple images per experimental group were taken and each experiment was repeated a minimum of three times. The number of complete meshes were manually quantified, and the number of junctions and tube length were quantified using the WimTube application from Wimasis [52].

### Cell growth assay

Cell proliferation was determined using the CellTiter-Glo® Luminescent Cell Viability Assay (Promega) as previously described [53]. Briefly, 1,000 cells were plated in a 96 well in 100 µL of full culture media. Cells were allowed to adhere and after 24 h, culture media was removed and cells received media with either CA3, Verteporfin or DMSO. The 0-h timepoint was assessed at this time. Subsequent time points were normalized to 0-h values to assess proliferation.

### In vivo melanoma models

#### Subcutaneous models

YUMMER1.7 (kind gift from Dr. Marcus Bosenberg, Yale University) or B16.F10 (ATCC Cat# CRL-6475) were trypsinized, washed twice with ice-cold PBS, and injected into the shaved flanks of wild-type 9 to11-week-old C57Bl/6 male mice at a density of 3 × 10^5^ cells in 100 μl of PBS. Male mice were used to avoid sex-related rejection of cells. Treatment with anti-VEGF and Lenvatinib began 7 days after subcutaneous injection, at which point the tumors were palpable. Mice were given intraperitoneal injections of 5 mg/kg anti-VEGF (clone G6-31, Absolute Antibody) or 10 mg/kg isotype control (Bio X Cell Cat# BE0089) twice weekly. Mice were treated with 10 mg/kg lenvatinib daily by oral gavage [54]. YUMMER1.7 injected mice were treated for 31 days and then monitored. B16.F10 injected animals were continually treated until the last mouse had to be euthanized due to tumor size > 1000 mm^3^ or tumor ulceration. Tumors were collected at sacrifice for histology.

#### Left ventricle model of melanoma metastasis

YUMMER1.7 or Cl.2A cells were engineered to express luciferase and directly injected into the left ventricle of Nair treated, wild-type C57Bl/6 8-week-old male mice or wild-type male nude mice, respectively, at 1 × 10^5^ cells in 100 μl of PBS as previously described [32, 54]. Expression of luciferase allows for tracking of metastases by bioluminescent imaging [55]. Immediately after LV injection, mice were injected with luciferin and IVIS-imaged to confirm systemic dissemination of cells. For the YUMMER1.7 model, the treatment regimen was the same as described in the subcutaneous model. For the Cl.2A model, treatment with 1–10 mg/kg CA3, 20 mg/kg TED-347 or vehicle was administered via intraperitoneal injection beginning one to three days after LV injection. Mice were treated 3 times a week for 13 weeks.

### Tissue histology

Immunohistochemical staining of CD34 and PAS and capturing of images was performed by Yale Pathology Tissue Services (YPTS). For YAP, TAZ and BIRC5 paraffin was removed from slides by three washes with xylene for 5 min, and rehydration in an ethanol gradient (100%, 100%, 95%, 80%, 70%) for 3 min each. Citrate antigen retrieval was performed in a pressure cooker for 15 min followed by cooling for 30 min under running water. Endogenous peroxidases were blocked in 0.3% hydrogen peroxide in deionized water followed by two 5-min washes with 1 × PBS. Slides were blocked with 2% horse serum in 0.1% Triton X in PBS for 1 h before an overnight incubation in a humid chamber at 4 °C with the primary antibody. The ImmPRESS HRP Horse anti-rabbit IgG PLUS Polymer Kit (Vector Labs, Cat# MP-7801) was used as per manufactures instructions. Slides were counterstained in hematoxylin and mounted with ProLong Gold (Thermo Fisher Scientific, Cat# P36934). Antibodies used are as follows: YAP (Cell Signaling Technologies, Cat.#14074; 1:1000), TAZ (Cell Signaling Technologies, Cat.#72804; 1:400), BIRC5 (Cell Signaling Technologies, Cat.#2808; 1:400) and IgG (Cell Signaling Technologies, Cat.#3900s).

### Statistical analysis

JMP Pro (16.2.0) software was used (SAS Institute, Cary, NC, USA) to analyze the VM and BV scoring determined using the TMA. VM and BV scores were dichotomized at the median. The Chi Square test was used for these dichotomized variables.

GraphPad prism software (version 8.0.2) was used to analyze the continuous scoring of BV and VM in relationship to tumor volume and edema via an unpaired two-tailed Student’s t-test. When analyzing differences between VM and BV and sites of metastasis (extracranial and intracranial), matched samples and a paired Student’s t-test was used. All in vitro experiments were completed a minimum of three times. For comparisons involving two experimental groups, an unpaired two-tailed Student’s t-test was used. A p-value of less than 0.05 was considered significant. All quantitative data are presented as mean ± SD. For in vivo statistics, survival analysis was assessed via Kaplan–Meier testing.

### Study ethical approval

All animal studies were approved by Yale University Institutional Animal care and Use Committee (IACUC) (Study # 2020-20152). Melanoma tumor(s) were collected with the approval of the Yale University Human Investigations Committee (Institutional Review Board, Yale HIC# 0609001869).

## Results

### Intracranial melanoma metastases display more vascular mimicry than extracranial metastases

**Fig. 1:**
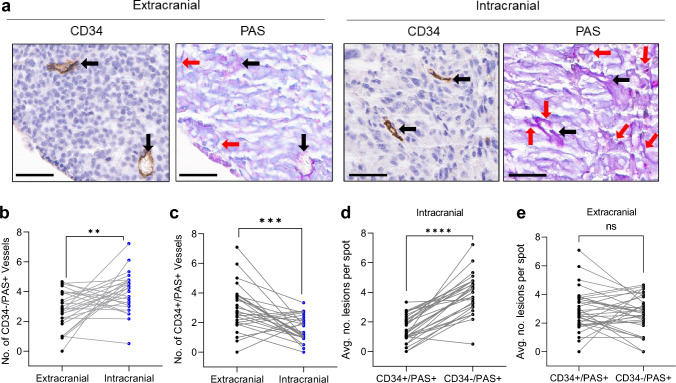
Intracranial melanoma metastases have higher levels of vascular mimicry than extracranial metastases: **a** Representative images of immunohistochemical staining for CD34 and PAS in extracranial and intracranial tumors isolated from patients at Yale University. Black arrow: CD34 + /PAS + , red arrow: CD34-/PAS + . Magnification of images are 20x with scale bars representing 50 µM. **b** Analysis of CD34-/PAS + vessels and CD43 + /PAS + vessels **c** in matched intracranial and extracranial metastasis patient samples. Black is intracranial, blue is extracranial, and the grey line connects the matched pairs. Analysis of CD34 + /PAS + and CD34-/PAS + vessels in intracranial metastases (**d**) and in extracranial metastases (**e**). The grey line connects values from the same tumor. Significance for all panels assessed by paired-Student’s t-test. ****p < 0.0001, ***p < 0.001, **p < 0.01

We previously showed that aggressive brain metastatic competent cells cultured in matrigel can readily induce vasculogenic mimicry (VM) in vitro, and VM competency seems to correlate with the ability of these cells to induce metastatic outgrowth [32]. Moreover, our previous studies also show that human resected melanoma brain metastases have lower CD34 + vessel density compared to matched extracranial metastases, but non-angiogenic vasculature has yet to be studied in specimens from patients with melanoma brain metastases [48]. We therefore sought to evaluate VM in matched cerebral and extra-cerebral metastases from a cohort of 37 melanoma cases previously described [48]. A specific feature of VM channels is a wall structure negative for CD34 staining but positive for PAS staining [32, 56, 57]. CD34 + /PAS + staining identifies endothelium-lined blood vessels (BV) whereas CD34-/PAS + staining identifies VM (Fig. 1a). Using methods we previously described [32], we conducted thorough microscopic examination of sections for CD34 and PAS staining, followed by quantification of BV and VM structures. We found a significantly higher number of VM structures in intracranial metastases compared to their matched extracranial counterparts (Fig. 1b). As expected, BV levels were higher in extracranial cases compared to matched intracranial cases (Fig. 1c). The same trends were found when our comparative analysis included all matched and unmatched tumor specimens on the TMA (Intracranial: 75 samples, Extracranial: 117 samples; Supplementary Fig. 1a, b). When intra-tumoral BV and VM structures were compared, we found that intracranial cases have significantly more VM than BV whereas there was no difference between the number of VM and BV in extracranial cases, and this was true whether analysis included only matched lesions (37 patients, Fig. 1d and e**,** respectively) or the entire cohort (Supplementary Fig. 1c and d, respectively). Collectively, these data suggest that intracranial melanoma metastases, but not extracranial melanoma metastases, may utilize VM over angiogenesis as a form of neovascularization in humans.

### Intracranial vascular mimicry is linked with MBM tumor volume and edema

**Fig. 2:**
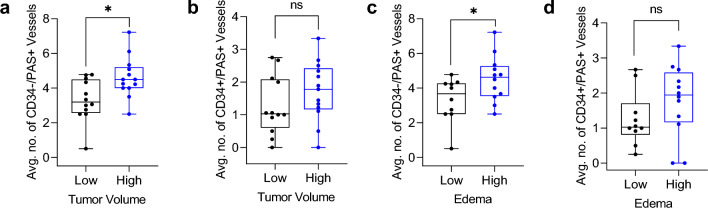
Vascular mimicry in melanoma brain metastases is associated with melanoma brain metastasis tumor volume and edema. **a** Two-sample unpaired t-test showing that high VM staining (continuous tumor CD34-/PAS+ scores) is significantly associated with high brain metastasis tumor volume (tumor volume scores dichotomized by the median value) (p = 0.0124). **b** Two-sample unpaired t-test showing that high blood vessel staining (continuous tumor CD3 + /PAS + scores) is not significantly associated with high brain metastasis tumor volume (tumor volume scores dichotomized by the median value) (p = 0.2402). **c** Continuous scores for VM staining (CD34-/PAS+) were plotted against intracranial edema scores dichotomized by the median value and significance assessed via two-sample unpaired T-test (p = 0.0415). **d** Two sample t-test of continuous BV scores (CD34+ /PAS+) and dichotomized edema scores (P = 0.1996)

Obtaining nutrients from the tumor microenvironment is important for tumor growth and viability, which is predominantly acquired via angiogenesis [12]. However, our group showed that there is no correlation between BV density and tumor volume in intracranial melanoma metastases [58]. Therefore, we sought to study the association between VM and brain tumor volume. When tumor volume was dichotomized by the median value into high and low groups, high tumor volume independently associated with high levels of VM in intracranial cases (Fig. 2a). However, as expected, there was no significant association between tumor volume and BV number (Fig. 2b*)*. These results were confirmed by Chi-square analysis of dichotomized VM (Supplementary Fig. 2a) or BV (Supplementary Fig. 2b) with continuous tumor volume scores. These results suggest that VM could facilitate brain metastasis tumor growth.

Using an alternative quantification method, we previously showed that brain tumor volume positively correlates with edema, derived by 3D modeling from patient MRIs [32] and this was once again confirmed in the current study by linear correlation (Supplementary Fig. 2c). Therefore, we further investigated the relationship between vasculature and edema, and found a statistically significant correlation between VM and increased edema (Fig. 2c). The same trend was seen for angiogenic vasculature, although the association between BV and high edema did not reach statistical significance (Fig. 2d). This suggests that VM could facilitate perilesional edema in patients with melanoma brain metastases.

### Brain metastatic cell lines form vascular structures in vitro

**Fig. 3:**
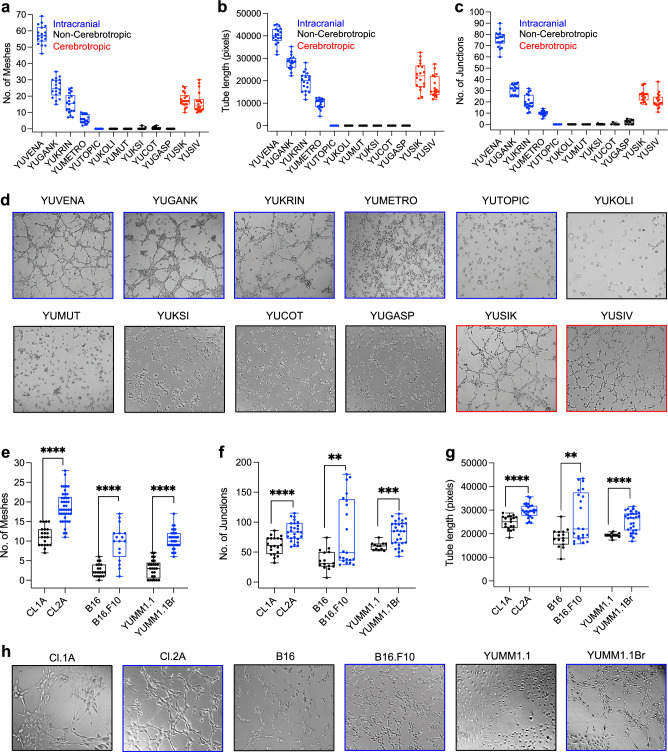
Melanoma cell lines form vascular networks in vitro. Quantification of the number of meshes (**a**), junctions (**b**) and tube length (**c**), in a melanoma cell line panel and the representative images (**d**). Blue represents short-term cultured patient derived cell lines from intracranial melanoma metastases, black represents short-term cultured patient derived cell lines from extracranial metastases, red represents short-term cultures derived from extracerebral metastasis in patients who developed overt brain metastases (cerebrotropic) (**d**; 10 × magnification). Quantification of the number of meshes (**e**), junctions (**f**) and tube length (**g**), in primary and brain metastatic cell lines. Black represents primary, blue represents cerebrotropic derivatives. B16 variants and YUMM1.1 variants are mouse cell lines. **h** Representative images of VM structures formed by the cell lines in panels **e**–**g** (10 × magnification). All data are expressed as mean $\pm$ SD of three biological replicates and statistical significance is determined using an unpaired Student’s T-test. ****p < 0.0001

To further study VM formation in advanced melanoma, we assessed the ability of several short-term human melanoma cell line cultures to form VM in vitro. Our assay includes cells derived from melanoma brain metastases (blue) and cells derived from extracranial metastases of patients who did not develop brain metastases (non-cerebrotropic, black) or from those who did (cerebrotropic, red). We found a tendency for cells established from melanoma brain metastases (YUVENA, YUGANK, YUKRIN, YUMETRO) to have a significantly better ability to induce VM in vitro compared to cells from other metastatic sites (YUKOLI, YUMUT, YUKSI, YUCOT, YUGASP) as determined by quantification of the number of meshes, junctions, and tube length (Fig. 3a–d). Similarly, YUSIK and YUSIV cells derived from extracranial specimens of patients who developed rapid and overt brain metastases, also exhibited high VM in vitro (Fig. 3a–d). Moreover, a pronounced ability to induce VM was seen in the brain metastatic mouse melanoma line derived from repeat left-ventricle injections and isolation of clones from the brain, YUMM1.1Br, compared to its parental counterpart, YUMM1.1, derived from a genetically engineered mouse model [50] (Fig. 3e–h). Similarly, the murine B16.F10 and human Cl.2A cell line derivatives which generate melanoma brain metastases [32, 59] exhibited VM as opposed to parental, non-brain metastatic B16 and Cl.1A cells, respectively (Fig. 3e–h). We also observed an increased amount of VM with mouse tumor endothelial cells as compared to normal endothelial cells (Supplementary Fig. 3). We did not see a significant difference in VM formation between YUMM1.7 and the mutagenized derivative, YUMMER1.7 (Supplementary Fig. 3). Lastly, in our previous study we identified paracrine stimulation of VM with Cl.2A cells [32]. Here, we found that conditioned medium from Cl.2A, YUSIK, and YUVENA cells enhance VM formation with the non-cerebrotropic cell lines, YUKOLI, YUKSI and YUCOT (Supplementary Fig. 4a–i). Additionally, we observed stimulation of VM formation by brain metastatic cells in the presence of endothelial cells in a co-culture model (Supplementary Fig. 5). Taken together, these studies indicate that VM is likely a process exploited to a greater extent in melanoma brain metastases.

### Targeting YAP/TAZ inhibits vascular mimicry in brain metastatic cell line derivatives

Recently, YAP/TAZ has emerged as a key mediator of VM [21, 29] and our previous work shows that altered YAP/TAZ signaling is associated with VM and tumor growth [32]. Here, we found YAP and TAZ to be expressed at variable levels in melanoma (Supplementary Fig. 3i, j) and that siRNA targeted inhibition of YAP, TAZ, and YAP/TAZ decrease VM formation in Cl.2A and YUSIK cells (Supplementary Fig. 6).

To further investigate YAP in human cerebral and non-cerebral melanoma tumors we employed quantitative immunofluorescence and a TMA of clinical samples from a historical cohort of metastatic melanoma cases with variable times to development of brain metastasis [49]. YAP activity was evaluated in a subset of these tumors (51 cases) for which transcript profiles were available from a previously published dataset [49]. The fluorescent signal was quantified within the S100-positive area or within the tumor DAPI positive nuclei. By two-way comparison, YAP levels in cerebral metastases were significantly higher when compared to extra-cerebral tumors suggesting that YAP expression might be associated with brain involvement of melanoma (Supplementary Fig. 7a, b). Consistent with these findings, examination of YAP distribution across various metastatic sites showed a prevalence of higher YAP in visceral metastases and low expression in skin lesions, further suggesting that YAP upregulation and activity could be associated with the location of the specific metastasis (Supplementary Fig. 7c, d). Furthermore, in this subset of cases, high YAP expression correlated with high VM, though data only trended toward significance (Supplementary Fig. 7e, f). Using a gene expression data set from our previously published tumor profiling studies, we found several known YAP/TAZ targets previously linked to angiogenesis to be differentially expressed in these metastatic tumors. For example, expression of DOCK5, RUNX1, ASAP1, JUN, FOS, positively correlated with YAP levels and for these comparisons p-values were for the most part significant or otherwise a trend was evident (Supplementary Fig. 7g). Interestingly, high YAP expression also correlated with downstream transcriptional targets previously implicated in VM such as SNAI2, an EMT regulator, and STAT3 and ANG2, two known potent proangiogenic factors (Supplementary Fig. 6h) [21, 60]. Additionally, CDC20 expression correlated with VM and a similar trend was seen for CCND1 and ANG2 genes (Supplementary Fig. 7h).

**Fig. 4:**
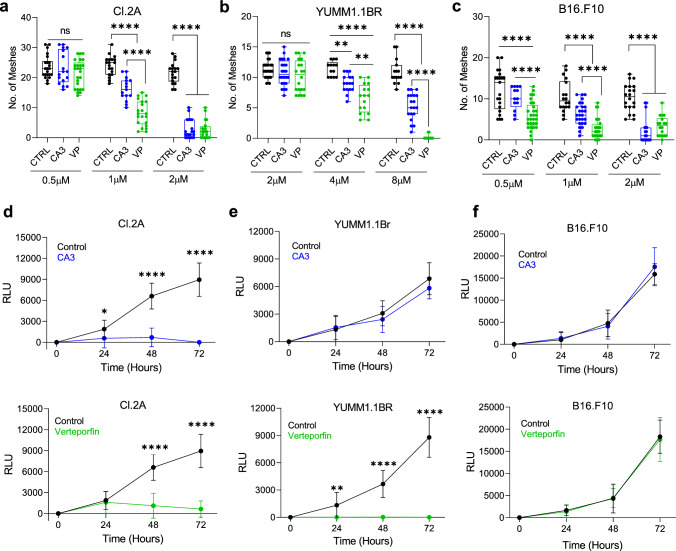
The effect of YAP/TAZ inhibitors on VM and growth in cerebrotropic derivatives of melanoma cell lines. CL.2A (**a**), YUMM1.1Br (**b**), and B16-F10 (**c**) cells were treated with increasing doses of CA3 and VP for 6 hrs at which point VM formation was assessed by mesh quantification. The number of meshes were counted for each treatment group. Black = DMSO, Blue = CA3, Green = VP. **d** CL.2A cells were treated with the active dose of CA3 (top, 1 µM) and VP (bottom, 1 µM), (**e**) YUMM1.1Br were treated with the active dose of CA3 (top, 4 µM) and VP (bottom, 4 µM), (**f)** and B16.F10 cells were treated with the active dose of CA3 (top, 1 µM) and VP (bottom, 0.5 µM), for 72 hrs and cell proliferation was assessed by Cell Titer Glo every 24 hrs. The 0 h timepoint represents cells assessed prior to drug treatment. Representative Relative Luminescent Units (RLU) are normalized by subtracting the 0 h values from subsequent measurements. All data are expressed as mean $\pm$ SD of at least three biological replicates and statistical significance is determined using an unpaired Student’s T-test at each time point. ****p < 0.0001, **p < 0.01, *p < *0.05*

To expand on siRNA targeted inhibition of YAP and TAZ we tested the effects of pharmacologically targeting YAP/TAZ with verteporfin (VP) and CA3, two known YAP/TAZ inhibitors, on VM formation in vitro, using the aggressive Cl.2A human melanoma cells and the two highly cerebrotropic derivatives of mouse melanoma cell lines, YUMM1.1Br and B16.F10 [61–65]. In Cl.2A, YUMM1.1Br, and B16.F10 cells we observe a dose dependent decrease of VM when treating with CA3 and VP when quantifying number of meshes (Fig. 4a–c) alongside number of junctions and tube length (Supplementary Fig. 8a–f). CA3 and VP inhibit VM in Cl.2A and YUMM1.1Br at 1 µM and 4 µM, respectively (Fig. 4a, b), and VP inhibits B16.F10 at 0.5 µM whereas the active dose for CA3 is 1 µM (Fig. 4c). Evaluation of live cell density using Cell Titer Glo shows no significant difference in cell viability, which is observed in all cell lines tested at the active dose of CA3 or VP at 6 hrs, the time of VM formation, an indication that drug effects on VM are not an indirect result of cell death (Supplementary Fig. 9a–d). At this timepoint, we observed a decrease in both YAP and TAZ protein levels upon treatment with VP, but not CA3 in Cl.2A, YUMM1.1Br, B16.F10, (Supplementary Fig. 10a–c). We also observed a variable decrease in select YAP/TAZ target genes after VP and CA3 treatments (Supplementary Fig. 11a–c). Using the lowest dose for VM inhibition for each tested cell line, we next assessed the effects of VP and CA3 on cell proliferation. In Cl.2A cells, there is significant growth inhibition at 1 µM CA3 and VP at 72 hrs (Fig. 4d). In YUMM1.1Br cells, 4 µM CA3 had no effect on proliferation after 72 hrs, while VP significantly inhibited cell proliferation (Fig. 4e). In B16.F10 cells, there was no significant difference in cell proliferation throughout the time course for the active dose of CA3 (1 µM) or VP (0.5 µM) (Fig. 4f). Lastly, we confirmed the importance of YAP/TAZ signaling in mediating VM through testing the effects of a third compound, TED-347. TED-347 is a covalent, allosteric inhibitor of the YAP1-TEAD4 protein–protein interaction which suppresses TEAD4 transcriptional activity [66]. TED-347 robustly inhibited VM in a dose dependent manner in Cl.2A cells (Supplementary Fig. 12a–d).

**Fig. 5:**
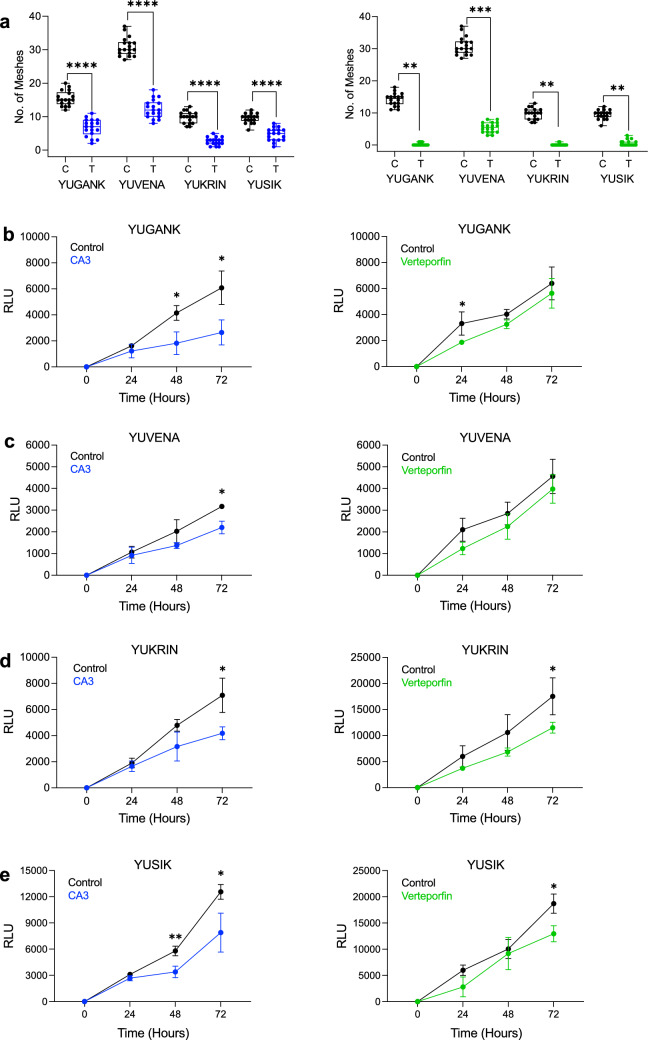
The effect of YAP/TAZ inhibitors on VM and growth in patient derived melanoma brain metastasis cultures. **a** YUGANK, YUVENA, YUKRIN and YUSIK were treated with 0.5 µM CA3 (blue, left) or 0.5 µM VP (green, right) for 6 h at which point VM formation was assessed by mesh quantification. YUGANK (**b**), YUVENA (**c**), YUKRIN (**d**) and YUSIK (**e**), were treated with the active dose of CA3 (0.5 µM) and VP (0.5 µM) for 72 hrs and cell proliferation was assessed by Cell Titer Glo every 24 hrs. The 0 h timepoint represents cells assessed prior to drug treatment. Representative Relative Luminescent Units (RLU) are normalized by subtracting the 0 h values from subsequent measurements. All data are expressed as mean $\pm$ SD of at least three biological replicates and statistical significance is determined using an unpaired Student’s T-test at each time point. ****p < 0.0001, ***p < *0.001,* **p < 0.01, *p < *0.05*

To confirm our results in short-term patient-derived cultures, we used YUGANK, YUVENA, and YUKRIN, cells derived directly from patient MBM, and YUSIK, obtained from an extracranial tumor specimen of a patient with brain metastasis. We found that the optimal dose for VM inhibition in these cell lines for both CA3 and VP is 0.5 µM (Fig. 5a, Supplementary Fig. 8 g–j). Notably, with this dose, there was no significant difference in cell viability observed in any cell line tested at 6 hrs, the time of VM formation (Supplementary Fig. 9 a and c), and at this timepoint we observed that CA3 and VP decreased both YAP and TAZ total levels in YUGANK, YUKRIN, and YUSIK cell lines (Supplementary Fig. 10e–g) alongside a variable decrease in select YAP/TAZ target genes (Supplementary Fig. 11d–g). Overtime CA3 inhibited proliferation of YUVENA, YUGANK, YUKRIN and YUSIK cells, but this effect was only significant after at least 48 hrs (Fig. 5b–e). By contrast, VP did not impact proliferation of YUGANK and YUVENA cells and only had a negligible inhibitory effect on proliferation in YUKRIN and YUSIK cells after 72 hrs (Fig. 5b–e). Collectively, these data suggest YAP/TAZ signaling might drive VM formation in melanoma brain metastasis while also mediating oncogenic growth, but this appears to be context dependent as the cell lines tested were affected differently by drug treatment.

### The effect of the YAP/TAZ inhibitor, CA3, on melanoma metastasis

**Fig. 6:**
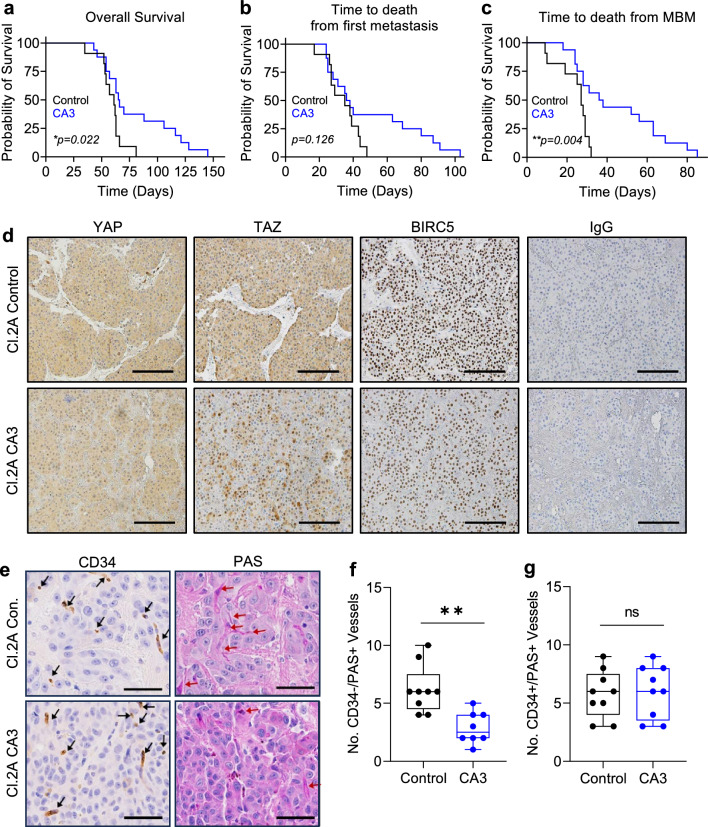
CA3 prolongs overall and brain metastasis survival in a murine model of metastasis. **a**–**c** Kaplan–Meier curves for mice receiving 1 mg/kg to 10 mg/kg treatment in a left ventricle injection murine model of brain metastasis. KM curves demonstrating the correlation between CA3 treatment and time overall survival (**a**, log-rank test, p = 0.022), the time to death from first metastasis diagnosis (**b,** log rank test, p = 0.126) and from brain metastasis diagnosis (**c,** log rank test, p = 0.004). **d** Representative images of immunohistochemical staining for YAP, TAZ, BIRC5, and IgG in Cl.2A brain tumors developed after left-ventricle injection in nude mice treated as controls, or with 1 mg/kg CA3. Scale bars represent 200 µM. **e** Representative images of immunohistochemical staining for CD34 and PAS in the same tumors. Black arrows point to BV, red arrows point to VM structures. Scale bars represent 50 µM. Quantification of VM (**f**; CD34-/PAS+) and BV (**g**; CD34+/PAS +) in nine areas from two tumors of each treatment group. Students t-test was used to assess significance between vascular density in control and CA3 treated mice. **p < *0.001*

Since YAP/TAZ pharmacologic inhibition attenuated cell growth and VM formation, we next sought to study its effects on metastatic growth employing a left-ventricle injection model of Cl.2A cells, which form both intracranial and extracranial metastases. Nude mice received 1 mg/kg–10 mg/kg CA3 three days after injection, prior to formation of any overt distant metastases. CA3 doses as low as 1 mg/kg have been shown to inhibit growth in non-metastatic models and is well tolerated [62]. All mice developed brain metastasis before death and treatment of CA3 was non-toxic to mice as assessed by weight (Supplementary Fig. 12e). We found that CA3 treatment prolonged overall survival as measured by the time to death from treatment initiation (Fig. 6a). Additionally, the time to death after detection of the first extracranial metastasis was delayed by CA3 treatment, though the difference between groups only trended toward significance (Fig. 6b). Interestingly, CA3 significantly prolonged the survival rate from date of first MBM detection (Fig. 6c). As expected, CA3 treatment decreased the levels of YAP, TAZ, and the target gene, BIRC5 (Fig. 6d). Moreover, CA3 treatment did not affect blood vessel density but decreased VM density in melanoma brain tumor specimens (Fig. 6e–g). We then sought to validate these data using TED-347 (20 mg/kg). TED-347 significantly prolonged mouse survival, time to death from first metastasis, and time to death from MBM (Supplementary Fig. 12f–h) and no drug related toxicity was observed (data not shown). Like CA3 treatment, TED-347 treatment did not affect blood vessel density but decreased VM density in melanoma brain tumor specimens (Supplementary Fig. 12i–k) These data suggest that drugs targeting YAP/TAZ could have activity in melanoma brain and extracranial metastases and enhance survival, which might be linked to their inhibitory effects on VM.

### Anti-angiogenic drugs do not inhibit vascular mimicry

**Fig. 7:**
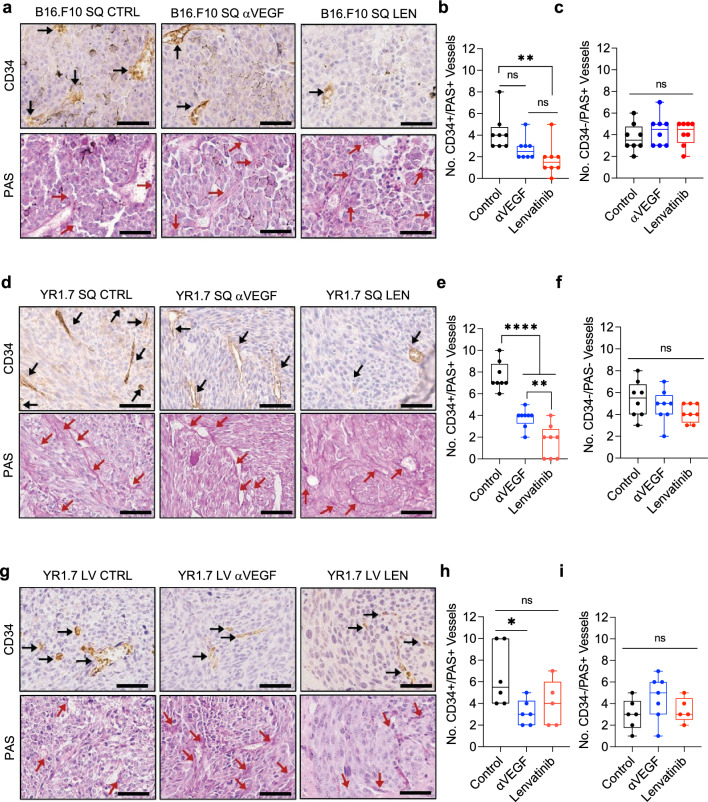
Assessment of VM in subcutaneous and brain metastatic mouse models of melanoma treated with anti-angiogenic drugs. **a** Representative images of immunohistochemical staining for CD34 and PAS in B16.F10 subcutaneous tumors harvested from C57BL/6 mice treated as controls, with 5 mg/kg αVEGF, or with 10 mg/kg lenvatinib. Black arrow: BV; CD34 + /PAS + , red arrow: VM; CD34-/PAS + . Magnification: 20×. **b** Quantification of the number of BV and **c** Quantification of VM vessels in three areas from two tumors of each treatment group. **d** Representative images of immunohistochemical staining for CD34 and PAS in YUMMER1.7 subcutaneous tumors harvested from C57BL/6 mice treated as controls, with 5 mg/kg αVEGF, or with 10 mg/kg lenvatinib. Black arrow: BV; CD34 + /PAS + , red arrow: VM; CD34-/PAS + . Magnification: 20×. **e** Quantification of the number of BV and VM (**f**) in three areas from two tumors of each treatment group. **g** Representative images of immunohistochemical staining for CD34 and PAS in YUMMER1.7 brain tumors developed after left-ventricle injection in C57BL/6 mice treated as controls, with 5 mg/kg αVEGF, or with 10 mg/kg lenvatinib **h** Quantification of the number of BV, VM (**i**) in three areas from two tumors of control and αVEGF, and one tumor from lenvatinib. All data are expressed as mean $\pm$ SD showing all points. All scale bars represent 200 µM. Statistical significance is determined using a one-way ANOVA ****p < 0.0001, **p < 0.001, *p < 0.05

Use of anti-angiogenic monotherapy in treatment of melanoma has failed to produce survival benefit [38], but anti-angiogenic therapies may have a role in advanced melanoma when used in combination with other drugs including immunotherapy (NCT03820986, NCT03776136) [39]. We therefore sought to study the effects of anti-angiogenic drugs currently used in the clinic on VM. Both subcutaneous xenograft and left-ventricle syngeneic mouse models were used to assess the effect of anti-VEGF (αVEGF), and the multiple tyrosine kinase inhibitor, lenvatinib on the formation of VM in vivo. C57BL/6 WT mice were subcutaneously injected with B16.F10 cells and treated with anti-VEGF, lenvatinib, or an IgG isotype starting 7 days after injection [54]. As expected, lenvatinib reduced the number of blood vessels identified by CD34 + /PAS + staining of subcutaneous murine tumor sections (Fig. 7a, b). The number of blood vessels were also reduced in anti-VEGF compared to control, but the difference only trended toward significance (Fig. 7a, b). Notably, the number of VM vessels (CD34-/PAS +), was comparable among the three groups (Fig. 7c). These results were confirmed using the YUMMER1.7 model where treatment with either anti-VEGF or lenvatinib significantly reduced the amount of CD34 + /PAS + vessels compared to control (Fig. 7d, e). As seen with the B16.F10 model, the number of CD34-/PAS + vessels were comparable among the three treatment groups in the YUMMER1.7 model (Fig. 7f). Moreover, these findings were further validated using the YUMMER1.7 left-ventricle metastasis model, where we similarly found a decrease in BV but not VM density with both anti-VEGF and lenvatinib treatments when compared to vehicle treated mice (Fig. 7g–i). These effects were next verified through in vitro VM assessment (Supplementary Fig. 13). As expected, we observed no significant difference in the number of VM meshes between treatments of YUMMER1.7 cells (Supplementary Fig. 13a). This result was further confirmed with YUMM1.1Br, Cl.2A, and B16.F10 cells (Supplementary Fig. 13b–d). These data suggest that highly metastatic melanoma cells might subvert inhibition of VEGF and perhaps the other targets of lenvatinib including FGFR, PDGFRα, KIT and RET to generate and maintain VM structures.

We next assessed the effects of a CA3, and lenvatinib, and the combination thereof in a subcutaneous model using YUSIK cells which were derived from an extracranial specimen of a patient who developed rapid and overt brain metastasis (Supplementary Fig. 14). We identified a trend in the reduction of tumor growth with CA3 treatment and lenvatinib treatment, but this did not reach statistical significance (Supplementary Fig. 14a). However, combined CA3 and lenvatinib therapy led to a significant decrease in tumor volume compared to control (Supplementary Fig. 14a). Quantification of blood vessel density showed a reduction with lenvatinib treatment when compared to both control and CA3 treatments (Supplementary Fig. 14b,d). VM density in CA3 and combination therapy compared to control was significantly decreased while no reduction in VM was observed in the lenvatinib treatment group compared with control (Supplementary Fig. 14c, d). Notably, no drug related toxicity was observed (Supplementary Fig. 14e). These data suggest that dual targeting of the YAP/TAZ and angiogenic pathways could be beneficial to limit primary tumor growth, as well as the intrinsic capacity to metastasize to the brain, though the influence of VM mediating the propensity of MBM at these early stages remain unknown.

## Discussion

Our current study provides insight into the clinical relevance and underlying mechanisms of vascular mimicry (VM) in melanoma brain metastases. VM is a form of non-angiogenic neovascularization generated by tumor cells, which is important in the early stages of tumor growth and micro-metastatic outgrowth, drug resistance, and ultimately patient overall survival [21, 29, 67]. Our lab previously showed that melanoma cells, which preferentially form brain metastases, generate VM structures likely driven by YAP/TAZ upstream regulators, FMOD and SOX2 [32], but the clinical relevance of VM in melanoma brain metastases and the role of YAP/TAZ signaling regulating this mechanism remains unknown. Here we aimed to further clarify the clinical relevance of VM and the involvement of YAP/TAZ as regulators of VM in melanoma brain metastasis.

Using samples from brain and extracranial metastases in the same patients, we find that VM vessels are more pronounced in intracranial metastases compared to other sites. We also found that in intracranial metastases, VM density is significantly higher than endothelium-lined vessel density. Our previous study demonstrated the uniqueness of the brain tumor microenvironment such that intracranial metastases are less vascularized when compared to extracranial metastases [48]. A previous study in glioblastoma reported that blood vessel density in VM-positive tumors is lower than in VM-negative tumors [68], a phenotype we also find true in melanoma brain metastases. These data suggest that VM in melanoma brain metastases could be an important mechanism used by melanoma tumor cells when angiogenesis is inadequate to overcome the highly non-permissive and poorly vascularized microenvironmental pressure of the brain.

We previously reported that melanoma brain metastasis tumor volume is positively associated with cerebral edema [58], but neither variable correlates with intracranial CD34 blood vessel density, which we confirm in the current study using an alternative quantification technique. Our new data also shows that elevated VM density in intracranial lesions is significantly associated with both high tumor volume and cerebral edema. We previously found a positive correlation between FMOD, a recently identified modulator of VM, and edema-to-tumor volume ratio derived by 3D modeling from patient MRI’s [32]. Furthermore, in an in vitro blood brain barrier (BBB) model, modulation of FMOD in brain metastatic melanoma cells altered endothelial barrier functions [32], which might translate to decreased microvascular perfusion and significant hypoxia, explaining our current finding that VM positively correlates with cerebral edema. In clinical settings, edema can cause severe morbidity or mortality in patients, and our study suggests VM might be linked to edema formation [58]. Though we did not find a significant association between edema, VM, and survival, we note our analysis was limited by the low number of cases for which clinical data was available for assessment. Additional studies of larger cohorts with patient brain metastasis are necessary to evaluate the association between CD34 + and VM vessel density, tumor volume, edema, and other clinical variables.

To further study VM in melanoma brain metastasis, we utilized short-term cell cultures derived from patient brain metastases, melanoma cells endowed with increased ability to metastasize to the brain (cerebrotropic), and non-brain metastatic counterparts. In an in vitro model of VM, we found that brain metastasis derived or cerebrotropic cell lines, but not their non-metastatic counterparts, generate VM structures when cultured alone. Therefore, VM may be an underlying cell program unique to a subpopulation of melanoma cells with competency to not only disseminate to the brain but also generate metastatic lesions. Moreover, in addition to intrinsic cellular mechanisms, our data suggest that at least in part, paracrine cellular mechanisms are also involved. As such, we found that non-cerebrotropic cells can generate VM in response to paracrine signaling from the microenvironment. Our previous studies investigated underlying cell mediators of VM and identified SOX2 and secreted FMOD [32]. SOX2 is involved in self-renewal and metastatic outgrowth and FMOD is an ECM component known to mediate cellular reprogramming [32]. Dual gene silencing of FMOD and SOX2 ablated VM and subsequent melanoma brain metastases. FMOD signals upstream of YAP/TAZ through FAK and LATS [32] while SOX2 may sustain YAP/TAZ signaling through a positive feedback mechanism [32, 69]. Interestingly, YAP/TAZ has emerged as a regulator of VM [31] and has implications in other tumor types [70]. Genetic silencing of YAP and TAZ decreased VM in cerebrotropic cells, indicating their important role in these cells. The clinical significance of YAP/TAZ dependent VM in brain metastases was highlighted by our analysis of patient tumors, which showed elevated YAP levels in brain metastases relative to other sites and tumors with high YAP expression more frequently displayed high VM. Nevertheless, our analysis of known downstream YAP/TAZ targets in profiled tumors revealed several genes that showed positive correlations with YAP/TAZ expression, which included SNAI2 which has important roles in VM [71–73], STAT3, a pro-angiogenic factor [73, 74], ANG2 which is involved in both angiogenesis and VM [75], and several others all previously linked to angiogenesis [76–81]. Since YAP/TAZ can induce angiogenesis through activation of multiple downstream targets, the same might be true in the case of VM regulation [21]. Interestingly, SNAI2 was previously linked to brain metastasis while activated STAT3 was shown to promote melanoma brain metastasis [82, 83]. Our data overall suggest that the co-activator function of YAP/TAZ may be relevant to melanoma brain metastasis. Future work is warranted to perform functional characterizations of these genes and to investigate their joint role in melanoma metastasis to determine if YAP/TAZ transcriptional control drives the development of brain metastasis, and if this converges at their function as VM regulators.

To investigate the impact of targeting YAP/TAZ on VM and melanoma brain metastasis we used verteporfin (VP) and CA3 and confirmed our findings with TED-347. VP is a FDA-approved photosensitizing agent used in the photodynamic treatment of ocular diseases including age-related macular degeneration which is characterized by abnormal blood vessel growth [61]. VP targets YAP signaling by directly binding to YAP and altering its confirmation to inhibit its interaction with TEAD [63]. CA3 has similar inhibitory effects on YAP/TAZ activity and protein levels but is slightly more effective than VP at lower drug levels and was not reported to induce toxicity in mice [62, 84]. TED-347 is an irreversible, covalent allosteric inhibitor of the TEAD-YAP protein–protein interaction and has anti-cancer properties [66]. In our brain metastatic cell lines, CA3 and VP effectively inhibited VM and decreased cell growth in vitro. An on-target effect was confirmed as VP and CA3 contribute to a decrease in total YAP or TAZ protein levels and show a decrease in YAP-TAZ regulated genes. Our in vitro findings corroborated observations from in vivo models, where pharmaceutical inhibition of YAP/TAZ resulted in a delay of tumor growth, which trended toward significance. Notably, the link between tumor growth and VM was also suggested by our study in patient tumors as a significant correlation was found between tumor volume and VM. Moreover, in our metastasis model, we found that YAP/TAZ inhibition resulted in enhanced survival and prolonged the time to death after brain metastasis detection while decreasing VM. This finding is particularly important as the development of overt MBM remains a predominant contributor to melanoma-related deaths [1–3]. Notably, targeting VM has just started to emerge as a therapeutic treatment strategy in the clinical setting. CVM-1118, a small molecule drug developed by TaiRx (inhibits Nodal, a TGFβ family protein) is being investigated in phase 2 trials as monotherapy in advanced neuroendocrine tumors (NCT03600233) or in combination with Nivolumab in hepatocellular carcinoma (HCC) (NCT05257590). Collectively, our data provide further evidence of the possible utility of YAP/TAZ inhibition as a strategy to target metastatic melanoma.

Our work leads us to speculate that VM may have an important role in vascularization of melanoma brain metastases where endothelial cell angiogenesis is minimal, suggesting these two processes might be compensatory in supporting tumor growth. Current angiogenesis inhibitors have little or no effect on VM and they can even induce VM by causing tumor hypoxia, explaining why VM was linked with resistance to anti-angiogenic treatment in melanoma [38, 40, 85] and other diseases [21, 29, 56, 68, 86]. Bevacizumab (anti-VEGF), and lenvatinib (kinase inhibitor of VEGF receptors VEGFR1-3, FGFR1-4, PDGFRα, Kit and RET [36, 37]) had no effect on VM in vitro with our brain tropic human and mouse melanoma cell lines. In a mouse model of melanoma brain metastases, bevacizumab or lenvatinib had a negligible effect on metastatic growth [54] and our current data shows that although both agents effectively reduce tumor associated angiogenesis, they have no effect on VM. This could have tremendous clinical significance and broad implications regarding the effectiveness of anti-angiogenic drugs currently used in clinic to treat melanoma. Further studies are necessary to determine if VM is associated with the previously noted lack of response or resistance to anti-angiogenic therapy in melanoma [38, 40, 85].

The YAP/TAZ signaling pathway has a central role in mediating resistance to major classes of cancer drugs [87]. VP mimics YAP knockdown to overcome resistance to RAFi, TKI or chemotherapy drugs [87]**.** Moreover, VP effectively restored BRAF inhibitor suppression and attenuated growth of resistant melanoma at low concentrations [88]. Similarly, CA3 induced anoikis in resistant melanoma cells and inhibited lung metastasis [89]**.** While direct inhibitors of YAP/TAZ are still under development, inhibition can also be achieved indirectly through targeting upstream or downstream signaling [87]. In endothelial cells VEGF and YAP/TAZ signaling converge as VEGF promotes YAP/TAZ activation, which contributes to angiogenesis [33]. However, when VEGF is inhibited, vasculogenesis which includes angiogenesis and VM, can be maintained via FAK dependent YAP/TAZ signaling, which we showed to be a mechanism employed in FMOD dependent VM [32, 33]. Collectively these studies suggest a link between vasculogenesis and YAP/TAZ which might mediate subsequent therapeutic resistance. Further studies are warranted to identify down-stream mediators of YAP/TAZ function, which might be easily druggable, providing new opportunities for developing better therapeutic strategies that co-target both VM and angiogenesis in metastatic melanoma.

While targeting VM has just started to emerge as a therapeutic strategy for cancer, there are currently no clinical trials testing direct YAP/TAZ inhibitors as transcriptional coactivators are difficult to target and possibly toxic [90]. New agents inhibiting YAP/TAZ functions have emerged and show acceptable toxicity profiles and potent anti- tumor effects in rodents [91–93]. Alternatively, co-treatment with other agents, particularly anti-angiogenic agents, may be a viable strategy to limit toxicity while overcoming resistance. The combination of the YAP/TAZ targeting agent, CA3, with lenvatinib in a subcutaneous model of a cell line derived from an extracranial site of a patient who developed rapid brain metastasis, slowed tumor growth and decreased VM density, providing pre-clinical rationale for future development of novel combination anti-vasculogenic therapies for these patients.

The data presented herein suggest that VM may be an important and targetable mechanism in melanoma and that VM inhibition might be most useful for treating MBM, an area of high unmet clinical need. We found that VM density is higher in MBM compared to extracranial melanoma metastases and that VM density, but not blood vessel density, is associated with tumor volume and edema in MBM. We also establish the utility of anti-YAP/TAZ therapy in mouse models of metastatic melanoma and note that treatment effectively inhibits VM and prolongs survival of mice with MBM, suggesting that targeting VM might be a promising strategy for treating MBM. Based on our findings, we conclude that VM formation and blood vessel density are independent and possibly compensatory mechanisms and further investigation of co-targeting VM and angiogenesis in MBM is warranted. A limitation of our study is the use of 2D culture systems to study VM. Additionally, as YAP and TAZ have widespread cellular effects, our data cannot not rule out the possibility that YAP/TAZ signaling sustains tumor growth in our models, and therefore the contribution of VM modulation to the effect of YAP/TAZ inhibition on brain metastasis and survival remains unclear. Efforts are ongoing in our laboratory to confirm these findings through using additional YAP pathway inhibitors with the combination of anti-angiogenetic drugs and incorporation of 3D culture systems in future studies, to better understand the balance of VM in sustaining MBM.

### Supplementary Information

Below is the link to the electronic supplementary material.

Supplementary file1 (PDF 4606 KB)

## Data Availability

Data sharing is not applicable to this article as no datasets were generated or analyzed during the current study. All data generated or analyzed during this study are included in this article and its supplementary files and are accessible from the corresponding author upon reasonable request.
