# Supplementary material for: Vascular mimicry as a facilitator of melanoma brain metastasis
Source: Cell Mol Life Sci. 2024 Apr 18;81(1):188. doi: 10.1007/s00018-024-05217-z (PMC11026261; doi:10.1007/s00018-024-05217-z)
Supplement: Supplementary file 1 — Supplementary file1 (PDF 4606 KB) [file 18_2024_5217_MOESM1_ESM.pdf]

SUPPLEMENTARY INFORMATION

Vascular mimicry as a facilitator of melanoma brain metastasis

Olivia K. Provance, PhD[1*], Victor O. Oria, PhD[2*], Thuy T. Tran, MD, PhD[1], Jasmine I. Caulfield, PhD[1], Christopher R. Zito, PhD[1,3], Adam Aguirre-Ducler[4], Kurt A. Schalper[4], Harriet M. Kluger, MD[1] and Lucia B. Jilaveanu, MD, PhD[1]

Affiliations:

[1]Department of Medicine, Section of Medical Oncology, Yale University School of Medicine, New Haven, CT, USA, [2]Biotech Research and Innovation Centre (BRIC), Faculty of Health and Medical Sciences, University of Copenhagen, 2200 Copenhagen, Denmark, [3]Department of Biology, School of Arts, Sciences, Business, and Education, University of Saint Joseph, West Hartford, CT, USA, [4]Department of Pathology, Yale University School of Medicine, New Haven, CT, USA

*Contributed equally to this work

[§]Correspondence should be addressed to:

Lucia Jilaveanu, MD, PhD

Section of Medical Oncology

333 Cedar Street, SHM234E

New Haven, CT 06520

Tel: (203) 737-8370, Fax: (203) 785-4116

E-mail: lucia.jilaveanu@yale.edu

Supplementary Methods

Tumor Microarray and Quantitative Immunofluorescence (QIF)

The tumor microarray that we have previously developed [1] was stained with YAP anti-rabbit monoclonal antibody (1:1000; clone D8H1X, Cat. #14074S, Cell Signaling Technologies). As previously described [2], antigen retrieval in sodium citrate was performed after de-paraffinization, and slides were incubated with primary antibody diluted in 0.3% BSA/1X TBS overnight at 4°C. Anti-rabbit horseradish peroxidase–decorated polymer backbone (Envision, Dako; Cat. #4003), and Cy5-tyramide (PerkinElmer Life Science Products; Cat. # SAT705A001EA) were used for target visualization. The melanoma tumor mask was generated using an anti-S100/HMB45 cocktail (S100: Abbomax Cat. #500-6824, Clone SPM354. HMB45: Biogenex Cat. #MU001A-UC) followed by incubation with the anti-mouse horseradish peroxidase–decorated polymer backbone (Envision, Dako; Cat. #4001), and Cy3-tyramide (Akoya Biosciences; Cat .#SAT704A001EA) were used for target visualization. Nuclei were stained with DAPI (Invitrogen; Cat #D1306) and slides were mounted with ProLong Gold antifade reagent containing 4′,6-Diamidino-2-phenylindole (DAPI; Invitrogen Cat. #P36931).

The TMAs were scanned using a multispectral Vectra Polaris instrument (Akoya Biosciences). Then, images were analyzed using the AQUA method of Quantitative immunofluorescence (QIF) (Navigate Biopharma, Carlsbad, California, USA). This analysis enabled objective and sensitive measurement of targets within user-defined tissue compartments (Camp et al, 2002). Briefly, the QIF score of YAP marker in all cells (DAPI+), the S100-positive tumor-cell compartment and the surrounding S100-negative non-tumor/stromal-cell tissue area was calculated by dividing the target pixel intensities by the area of S100-positive or S100-negative pixels. This allows for comparisons across cases with dissimilar tumor and stromal content. The fluorescence scores obtained are normalized by the exposure time and bit depth, making them comparable across cases. Methods are also described previously [3].

Conditioned medium vascular mimicry

Cl.2A, YUSIK, and YUVENA cells were plated in a 10-cm dish and were serum starved for 24 hours. Conditioned medium was collected the day of the VM assay. 100μl chilled Matrigel (Corning) was added to a chilled 48-well plate and incubated for at least 30 minutes at 37°C to allow Matrigel polymerization. 30,000 YUKOLI, YUKSI or YUCOT melanoma cells in 300μL were seeded dropwise to each well in reduced serum media as the control or conditioned medium from Cl.2A, YUSIK or YUVENA. Images were taken 4 hours later and analyzed.

Vascular mimicry with endothelial cell co-culture

MS1 and bEnd.3 cells were plated in full culture medium in a 0.4μM pore insert fit for a 24-well plate and allowed grow overnight. 200μl chilled Matrigel (Corning) was added to the empty wells of the 24-well plate and incubated for at least 30 minutes at 37°C to allow Matrigel polymerization. 0.4μM pore inserts were moved to the wells with the Matrigel and 60,000 B16 or B16.F10 cells in 600μM reduced serum media were plated dropwise on the Matrigel. VM was imaged and analyzed after 24 hours.

Vascular mimicry with Lenvatinib and anti-VEGF treatment

Vascular mimicry assay was completed as described previously [4]. 100μl chilled Matrigel (Corning or R&D) was added to a chilled 48-well plate and incubated for at least 30 minutes at 37°C to allow Matrigel polymerization. 30,000 melanoma cells in 300μL were seeded dropwise to each well in 2% serum culture media apart from the YUMM lines being seeded in full media. Lenvatinib, mouse anti-VEGF, or human anti-VEGF (bevacizumab) were added to the mixture of cells prior to adding dropwise to the well. The final concentration of lenvatinib (LC Laboratories) used for treatment was 10μM and anti-VEGF was 1mg/mL. Mouse anti–VEGF was purchased

from Absolute Antibody (clone G6-31, Cat# 1022-2.0) and human anti-VEGF (bevacizumab; MVASI) was purchased from Amgen (NDC SS513-206-01).

Endothelial tube formation assay

The in vitro angiogenesis assay kit (ECM640-Millipore) was used. Briefly, 50 µl of chilled ECMatrix™ Gel Solution was added to multiple wells of a chilled 96-well plate and incubated at 37°C for at least 30 min to allow polymerization. Normal endothelial cells (NEC) and tumor endothelial cells (TEC) were trypsinized and counted, and 6,000 cells in serum-free endothelial cell medium were added to each well. Tube formation was quantified by the ImageJ angiogenic analyzer. b.End5 was used as the TEC. b.End5 is an endothelial cell line isolated from brain tissue of a Balb/c mouse with endothelioma. This was a gift from the depositor (Culture Collection.org.uk, Cat# 96091930) to Dr. Rafael Reuten at the University of Copenhagen. NEC is a normal endothelial cell derived from a healthy brain of a Balb/C mouse (Cell Biologics, Cat# BALB-5023).

Small interfering RNA (siRNA) transfections

Cl.2A and YUSIK cells were seeded overnight and transfected at 60-80% confluency with 60-100nM of targeted pooled siRNAs against YAP (Santa Cruz Biotechnology: sc-38637), TAZ(WWTR1) (Santa Cruz Biotechnology #sc-38568), or with a scrambled RNA (siControl; Santa Cruz Biotechnology, sc-37007) introduced by Lipofectamine 2000™ (Invitrogen CAT#1668019) in OptiMEM reduced-serum medium (Gibco CAT#11058-21). After overnight incubation the transfection mixture was replaced with normal culture medium. 48 hours post-transfection cells were harvested as normal and subjected to the in vitro vascular mimicry assay as described previously [4].

Western Blot

For western blots, cells were lysed using RIPA solution supplemented with 1mM Na_3VO_4, 1mM PMSF, and 1M NAF containing the protease inhibitor cocktail. Protein concentrations of lysates was determined Bicinchoninic Acid (BCA) assay. 20-30 µg of protein was diluted in a sample buffer (4X Laemmli Buffer: Bio-Rad supplemented with 1part β-mercaptoethanol) and subjected to sodium dodecyl sulfate-polyacrylamide gel electrophoresis (SDS-PAGE). Detection of proteins was done with HRP-conjugated anti-mouse or anti-rabbit IgG secondary antibodies (#7076 and #7074 respectively: Cell Signaling). Antibodies used in this study are in the table below:

Primary Antibody	Cat. No.	Company	MW (kDa)	Dilution	2° Antibody
YAP/TAZ D24E4	#8418	CST	50 & 70	1:1000	Anti-Rabbit
YAP1	#14074S	CST	65-78	1:1000	Anti-Rabbit
Phospho-YAP S127 (D9W2I)	#13008	CST	65-75	1:1000	Anti-Rabbit
Phospho-TAZ S89	#4911	CST	55	1:1000	Anti-Rabbit
β-Actin	A1978	Millipore Sigma	42	1:10,000	Anti-Mouse

Quantitative RT-PCR

Quantitative RT-PCR was performed as described previously [4]. Total RNA was extracted from cultured cells using RNeasy Kit (Qiagen) followed by reverse transcription using the iScript™ cDNA Synthesis Kit (Bio-Rad). Quantitative PCR was performed using Power SYBR® Green PCR Master Mix (Applied Biosystem/Thermo Fisher Scientific, Life Technologies). β-actin levels were used as an endogenous control for normalization and data analysis was performed using the comparative threshold cycle (CT) method. The following primers were used:

Gene	Forward Primer	Reverse Primer
ACTIN (h)	GATTCCTATGTGGGCGACGA	TCTCCATGTCGTCCCAGTTG
BIRC5 (h)	TCAAGGACCACCGCATCTCT	TGTTCCTCTATGGGGTCGTCA
CDC20 (h)	GCCGCCTTGTGAAAGAAACC	CTGACTGGTTCGGGGGATTT
CTGF (h)	GGTGTGGCTTTAGGAGCAGT	TGATGGCTGGAGAATGCACA
CYR61 (h)	GCCGCCTTGTGAAAGAAACC	CTGACTGGTTCGGGGGATTT
ZEB2 (h)	CAACCATGAGTCCTCCCCAC	GTCTGGATCGTGGCTTCTGG
ACTIN (m)	GGCTGTATTCCCCTCCATCG	CCAGTTGGTAACAATGCCATGT
BIRC5 (m)	CCTACCGAGAACGAGCCTGATT	CCATCTGCTTCTTGACAGTGAGG
CDC20 (m)	GATGGACGACATCTGGCAAGTG	GTTGCCAGGATATTGGACTGCC
CTGF (m)	GGGCCTCTTCTGCGATTTC	ATCCAGGCAAGTGCATTGGTA
CYR61 (m)	CTGCGCTAAACAACTCAACGA	GCAGATCCCTTTCAGAGCGG
ZEB2 (m)	CCACGCAGTGAGCATCGAA	CAGGTGGCAGGTCATTTTCTT

Subcutaneous tumor growth

YUSIK cells were trypsinized, washed twice with ice-cold PBS and injected into 6-10 week-old nude male mice at a density of 3 x 10^5 cells in 100 µl of PBS: Matrigel (1:1 ratio) into one hind flank of nude mice (n = 5 per group). Tumor growth was measured every three days using a digital caliper and tumor volume estimated using the formula ($V = L \times (W^2)/2$) as described previously [4]. Beginning the day before subcutaneous injection of tumor cells, mice were treated with vehicle, 10mg/kg lenvatinib, 10mg/kg CA3, or a combination of lenvatinib and CA3 at the respective doses. Mice received lenvatinib treatment daily, and CA3 treatment 3x/week.

Supplementary References

1. Jilaveanu, L.B., et al., *PLEKHA5 as a Biomarker and Potential Mediator of Melanoma Brain Metastasis.* Clin Cancer Res, 2015. **21**(9): p. 2138-47.
2. Zhang, H., et al., *PLEKHA5 regulates tumor growth in metastatic melanoma.* Cancer, 2020. **126**(5): p. 1016-1030.
3. Camp, R.L., G.G. Chung, and D.L. Rimm, *Automated subcellular localization and quantification of protein expression in tissue microarrays.* Nat Med, 2002. **8**(11): p. 1323-7.
4. Oria, V.O., et al., *Coupled fibromodulin and SOX2 signaling as a critical regulator of metastatic outgrowth in melanoma.* Cell Mol Life Sci, 2022. **79**(7): p. 377.

Supplementary Table 1

Human melanoma cell lines							
Cell line	Gender	Stage	Primary Lesions	Origin of cell line	BRAF	NRAS	Citation
YUVENA	M/53	IV	Unknown	Brain	V600R	WT	
YUGANK	M/78	IV	Cheek, face, head	Brain	WT	Q61K	
YUKRIN	F/37	IV	Left back, trunk	Brain	WT	WT	
YUMETRO	M/71	III	Back	Brain	WT	Q61L	1
YUTOPIC	F/56	IV	Back	Brain	V600E	WT	
YUKOLI	M/53	IV	Right pectoral region	Right axillary lymph node	V600E	WT	2
YUMUT	M/44	III	Cutaneous melanoma	Wrist, soft tissue	V600E	WT	
YUKSI	M/62	IV	Right temple, scalp	Right neck	V600K	WT	3
YUCOT	F/33	IV	Right neck	Ulna metastases	V600E	WT	
YUGASP	F/88	III	Cutaneous melanoma	Lymph node	WT	Q61L	
YUSIK	F/49	IV	Right thigh	Right inguinal lymph node	V600E	WT	1,2
YUSIV	F/61	IV	Right lower back	Right chest	WT	WT	2,3
YUGEN8	F/44	IV		Brain	V600E	WT	2,3

Mouse Melanoma Cell Lines					
Cell line	Gender	Mouse Strain	BRAF	NRAS	Citation
B16	M	C57BL/6J	WT	WT	4
B16.F10	M	C57BL/6J	WT	WT	4
YUMM1.1	M	C57BL/6J	V600E	WT	5
YUMM1.1BR	M	C57BL/6J	V600E	WT	
YUMM1.7	M	C57BL/6J	V600E	WT	5
YUMMER1.7	M	C57BL/6J	V600E	WT	6

1. Krauthammer M, Kong Y, Ha BH, Evans P, Bacchiocchi A, McCusker JP, Cheng E, Davis MJ, Goh G, Choi M, Ariyan S, Narayan D, Dutton-Regester K, et al. Exome sequencing identifies recurrent somatic RAC1 mutations in melanoma. Nat Genet. 2012; 44:1006-14

2. Halaban R, Zhang W, Bacchiocchi A, Cheng E, Parisi F, Ariyan S, Krauthammer M, McCusker JP, Kluger Y, Sznol M. PLX4032, a selective BRAF(V600E) kinase inhibitor, activates the ERK pathway and enhances cell migration and proliferation of BRAF melanoma cells. Pigment Cell Melanoma Res. 2010 Apr;23(2):190-200. doi: 10.1111/j.1755-148X.2010.00685.x. Epub 2010 Feb 10. Erratum in: Pigment Cell Melanoma Res. 2012 May;25(3):402. PMID: 20149136; PMCID: PMC2848976.

3. Krauthammer M, Kong Y, Bacchiocchi A, Evans P, Pornputtapong N, Wu C, McCusker JP, Ma S, Cheng E, Straub R, Serin M, Bosenberg M, Ariyan S, et al. Exome sequencing identifies recurrent mutations in NF1 and RASopathy genes in sun-exposed melanomas. Nat Genet. 2015; 47:996-1002.

4. Melnikova VO, Bolshakov SV, Walker C, Ananthaswamy HN. Genomic alterations in spontaneous and carcinogen-induced murine melanoma cell lines. Oncogene. 2004 Mar 25;23(13):2347-56. doi: 10.1038/sj.onc.1207405. PMID: 14743208.

5. Meeth K, Wang JX, Micevic G, Damsky W, Bosenberg MW. The YUMM lines: a series of congenic mouse melanoma cell lines with defined genetic alterations. Pigment Cell Melanoma Res. 2016 Sep;29(5):590-7. doi: 10.1111/pcmr.12498. Epub 2016 Aug 3. PMID: 27287723; PMCID: PMC5331933.

6. Wang J, Perry CJ, Meeth K, Thakral D, Damsky W, Micevic G, Kaech S, Blenman K, Bosenberg M. UV-induced somatic mutations elicit a functional T cell response in the YUMMER1.7 mouse melanoma model. Pigment Cell Melanoma Res. 2017 Jul;30(4):428-435. doi: 10.1111/pcmr.12591. Epub 2017 Jun 8. PMID: 28379630; PMCID: PMC5820096.

Supplementary Fig. 1

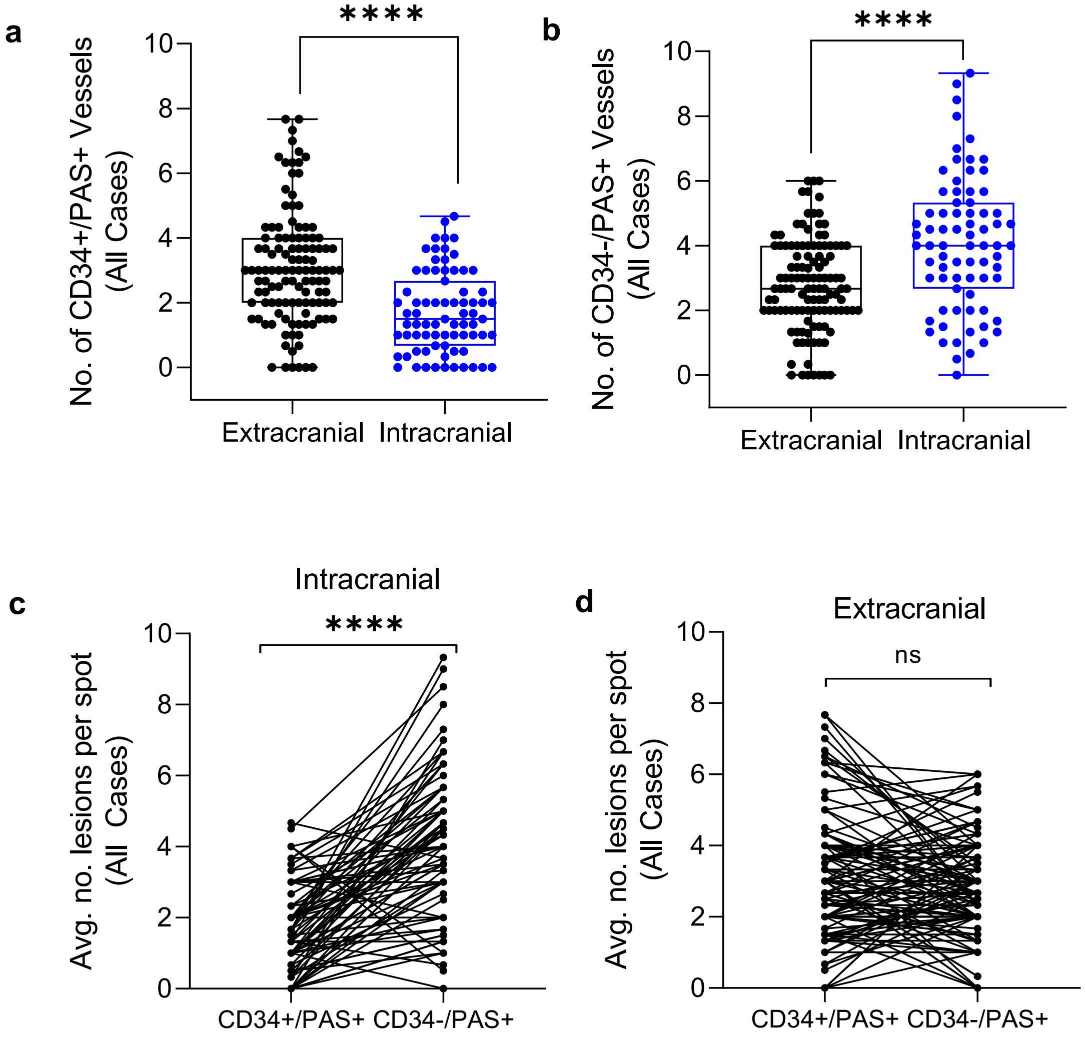

Supplementary Fig. 1

Associations between vascular mimicry and metastatic site. **a**: Analysis of CD34+/PAS+ vessels in all intracranial and extracranial spots included in our TMA. **b**: Analysis of CD34-/PAS+ in all intracranial and extracranial spots included in our TMA. **c-d:** Analysis of CD34-/PAS+ and CD34+/PAS+ vessels in all intracranial tumor spots (75 spots total) (**c**) and in extracranial tumor spots (117 spots total) (**d**). The black line connects values from the same tumor. Significance for panels a-b assessed by unpaired T-test. ****p<0.0001 and significance for panels c-d assessed by paired-Student's t-test. ****p<0.0001.

Supplementary Fig. 2

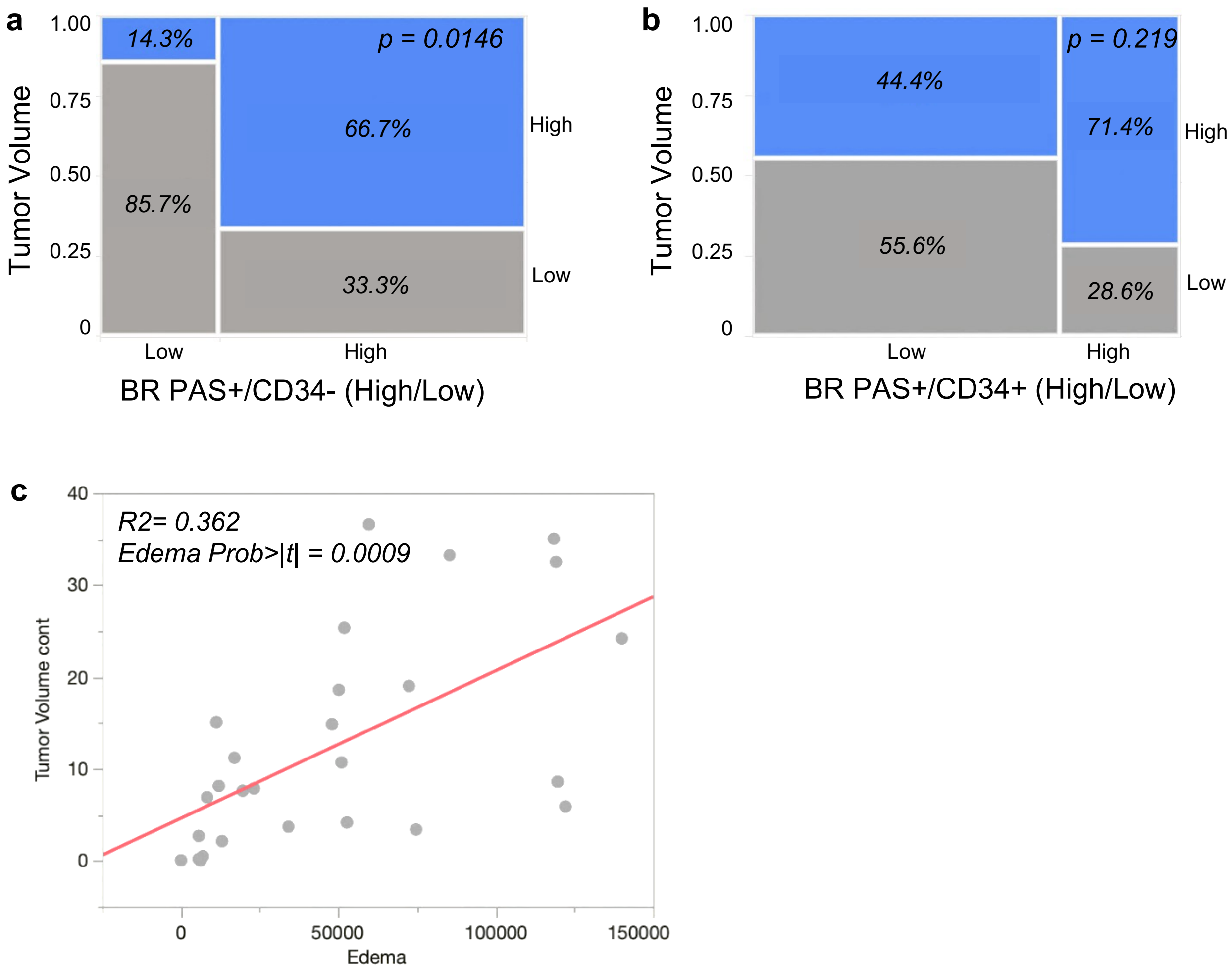

Supplementary Fig. 2
The association of tumor volume, edema, and vascular mimicry in melanoma brain metastasis. **a**: Chi-squared analysis comparing tumor volume with VM (**a**) or BV (**b**) in brain metastatic samples (p=0.0146 and p=0.219 respectively). **c**: Linear regression of continuous tumor volume scores and continuous edema scores. Edema and tumor volume have a significant positive correlation (r^2 = 0.362).

Supplementary Fig. 3

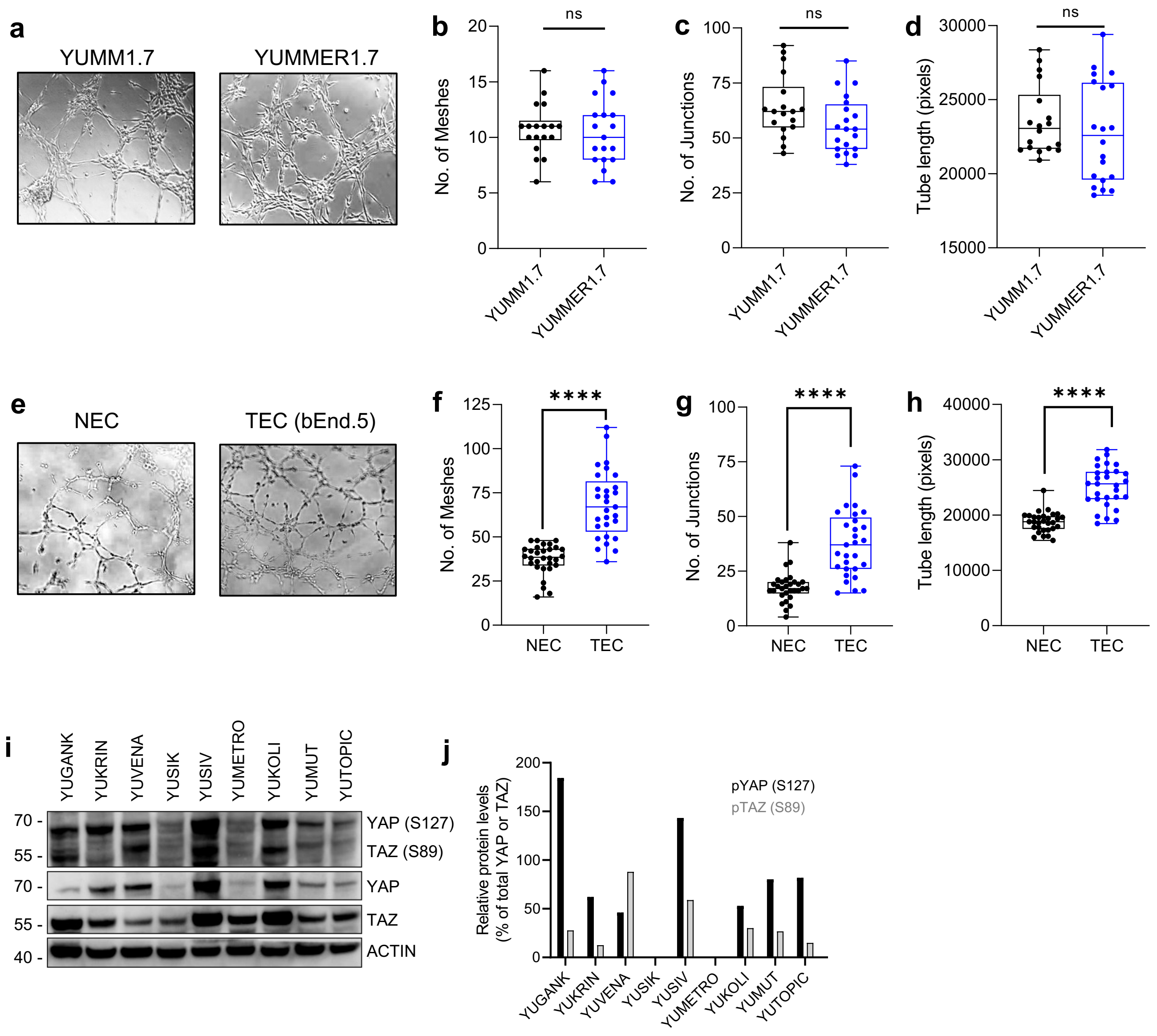

Supplementary Fig. 3

Vascular mimicry in YUMM1.7 variants. **a**: Representative images of VM formation in YUMM1.7 and YUMMER1.7 cells at 6 hours in full media taken at 10x magnification. Quantification of the number of meshes (**b**), junctions (**c**), and tube length (**d**) in YUMM1.7 and YUMMER1.7 cells using the representative images. **e**: Vascular mimicry in mouse normal endothelial cells (NEC) and mouse tumor endothelial cells (TEC, bEnd.5). **f-h:** Quantification of the number of meshes (**f**), junctions (**g**), and tube length (**h**). All data are expressed as mean ± SD of three biological replicates and statistical significance is determined using an unpaired Student's T-test. **i-j:** Representative western blot of phospho-YAP, phospho-TAZ, YAP and TAZ expression in a panel of human melanoma cell lines with quantifications of pYAP and pTAZ.

Supplementary Fig. 4

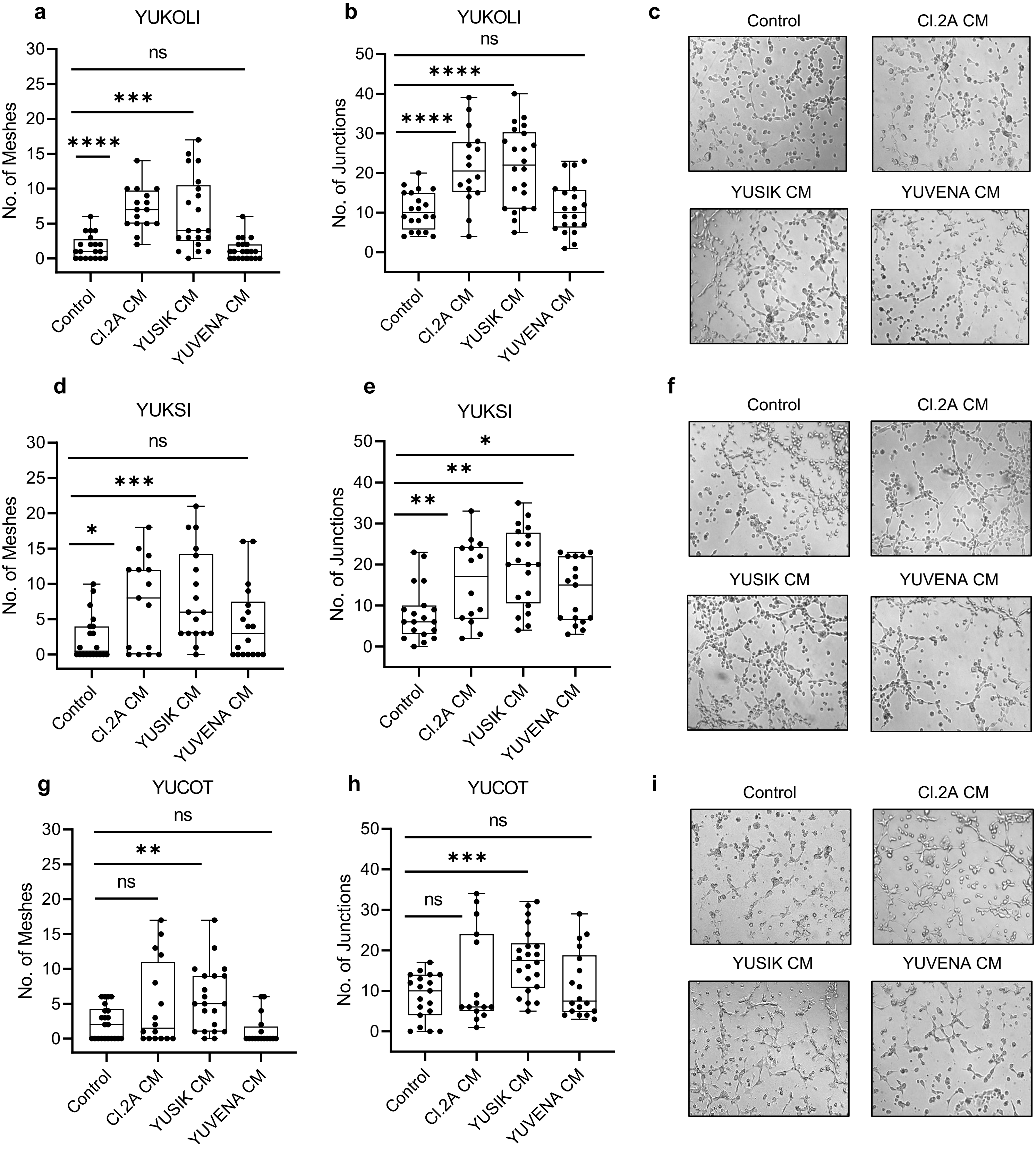

Supplementary Fig. 4

Paracrine induction of VM in vitro. Quantification of the number of meshes, number of junctions, and tube length (pixels) followed by representative images (10x magnification) in non-cerebrotropic cell lines (YUKOLI (**a-c**), YUKSI (**d-f**), and YUCOT (**g-i**)) cultured in conditioned media from Cl.2A, YUSIK, or YUVENA for 4 hours. All experiments were completed under serum starved conditions. All data are expressed as mean ± SD of three biological replicates and statistical significance is determined using an unpaired Student's T-test. *p<0.05, **p<0.01, ***p<0.001 ****$p<0.0001$

Supplementary Fig. 5

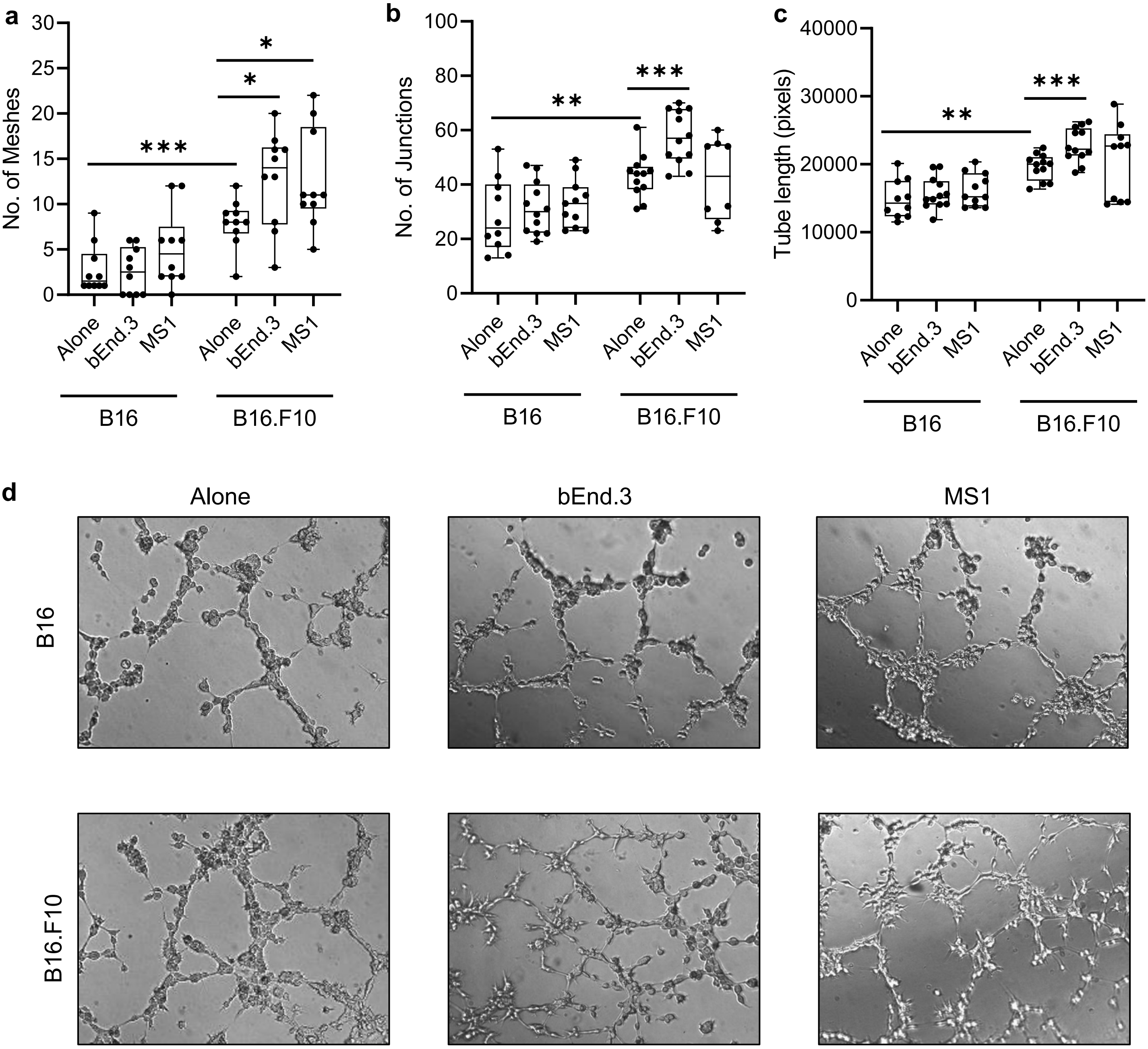

Supplementary Fig. 5
Endothelial cell induction of cancer cell VM in vitro. Mouse melanoma cells (B16 and B16.F10) were indirectly co-cultured with mouse endothelial cells bEND.3 and MS1. **a-c:** Quantification of the number of meshes, number of junctions, and tube length (pixels) of B16 and B16.F10 cells. **d**: Representative images (10x magnification) in B16 and B16.F10 cells under co-culture conditions after 24-hours. All data are expressed as mean ± SD of two biological replicates and statistical significance is determined using an unpaired Student's T-test. *p<0.05, **p<0.01, ***p<0.001 ****$p<0.0001$

Supplementary Fig. 6

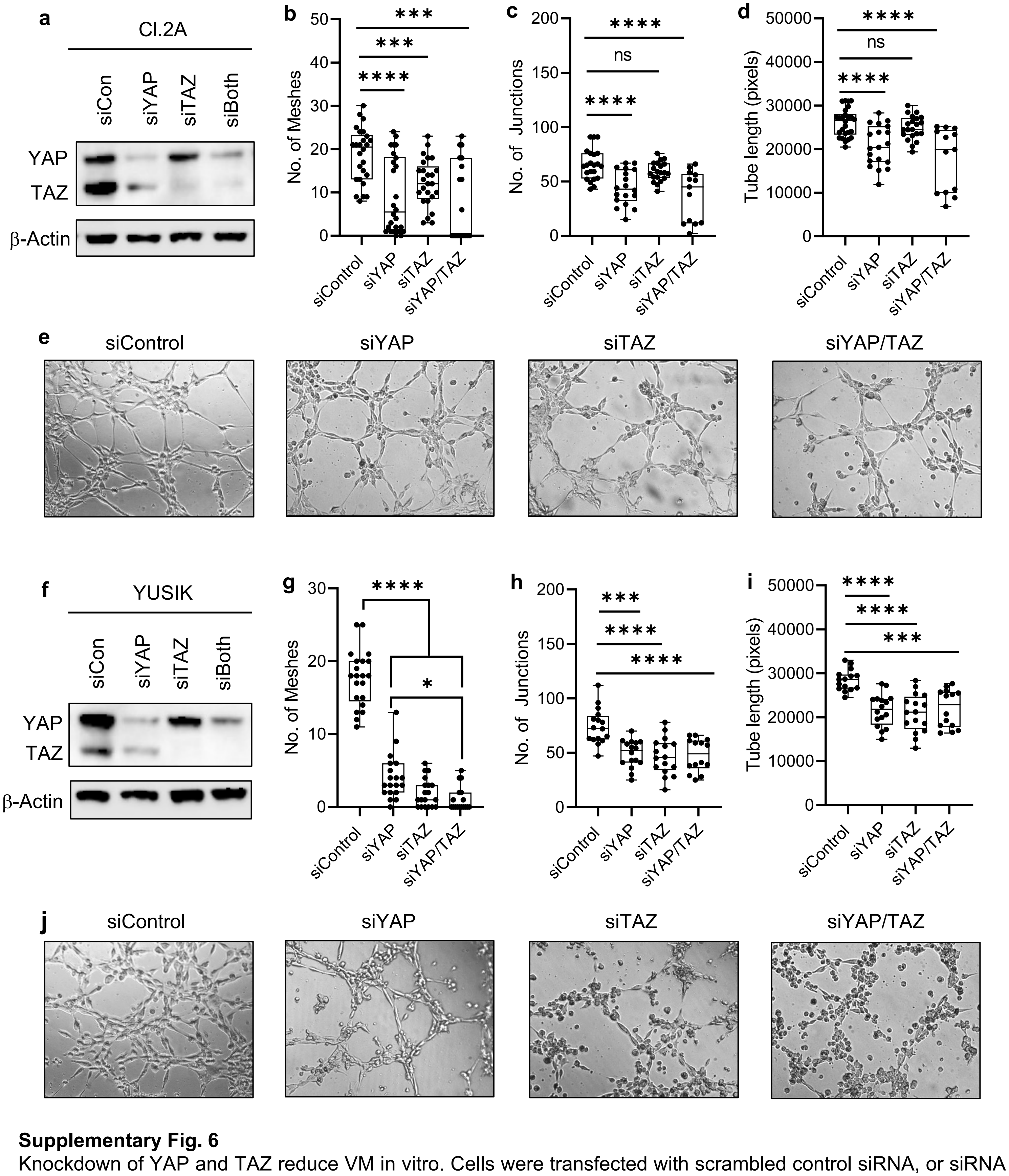

Supplementary Fig. 6
Knockdown of YAP and TAZ reduce VM in vitro. Cells were transfected with scrambled control siRNA, or siRNA against YAP, TAZ, or the combination 48-hours before cells were harvested and plated on Matrigel for the VM assay (see Materials and Methods). VM was completed in serum starved media with 2% FBS. Western blot of Cl.2A (**a**) and YUSIK (**f**) depicting YAP and TAZ knockdown with siRNA after 48 hours. Quantification of the number of meshes, number of junctions, and tube length in Cl.2A (**b-d**) and YUSIK (**g-i**) followed by representative images (Cl.2A (**e**) and YUSIK (**j**) (10x magnification)) after 4-hours. All data are expressed as mean ± SD of three biological replicates and statistical significance is determined using an unpaired Student's T-test. *p<0.05, **p<0.01, ***p<0.001 ****$p<0.0001$.

Supplementary Fig. 7

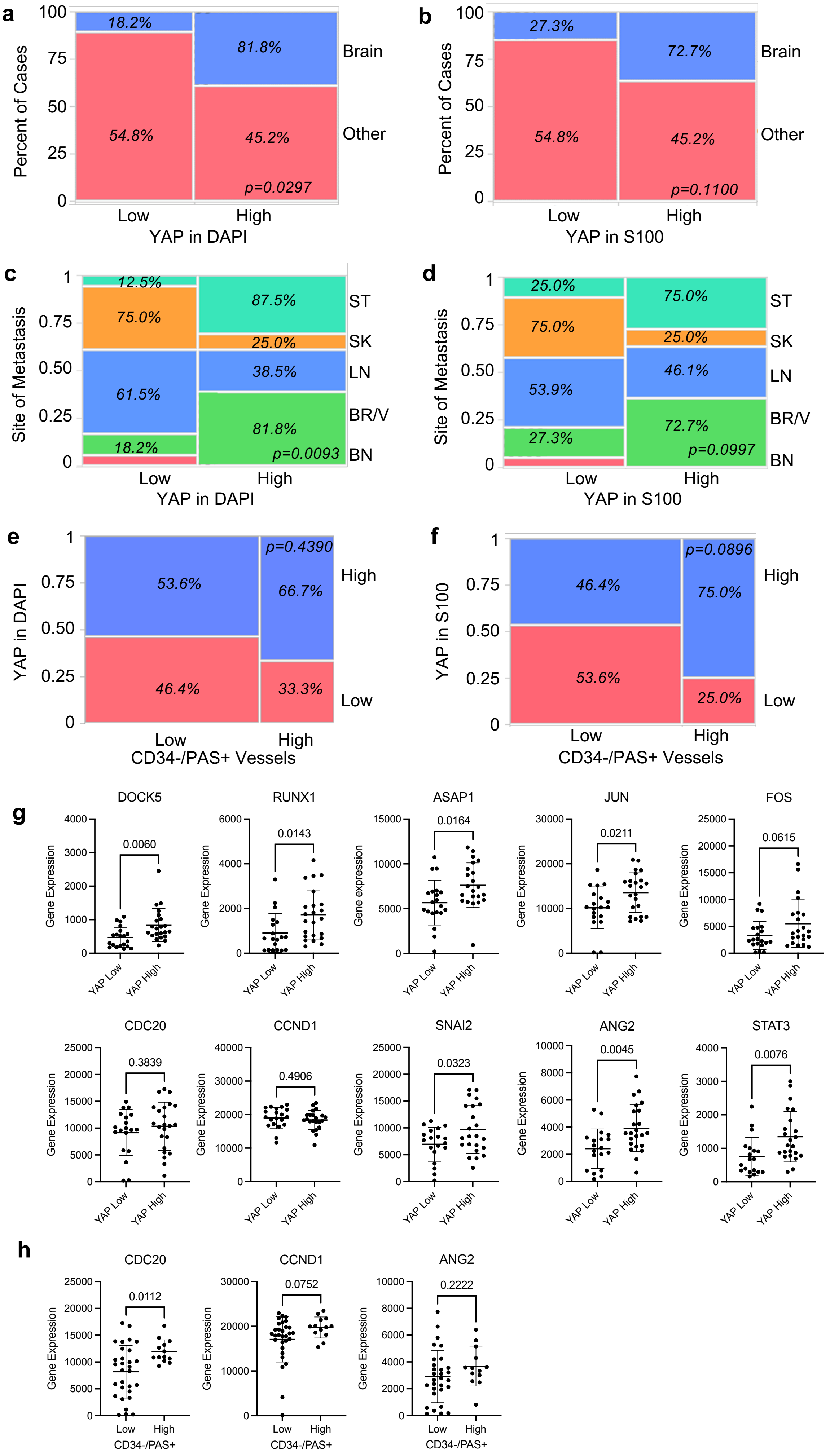

Supplementary Fig. 7

Analysis of YAP expression in samples from brain metastatic melanoma patients (**a-b**). YAP fluorescent signal in DAPI-positive nuclei (**a**) and S100-positive tumor cells (**b**) was dichotomized into high/low groups by the associated median value and assessed in brain metastases and extracranial metastases. There were significantly more cases of brain metastases that had high YAP compared to low YAP in the DAPI compartment (**a**; Chi-square, p = 0.0296) and the same trend was observed in S100 compartment (**b**; Chi-square, p = 0.11). (**c**) Examination of YAP distribution across various metastatic sites showed a prevalence of higher YAP signal in the DAPI compartment in brain metastases and low YAP signal in skin lesions, compared to all other metastatic sites (**c**; Chi-square, p = 0.0093 ST: Soft tissue, SK: Skin, LN: Lymph node, BR/V: Brain/Visceral, BN: Bone). The same trend was observed in the S100 compartment (**d**; Chi-square, p = 0.0997). (**e-f**): YAP expression in both the DAPI (**e**) and S100 compartment was compared between high or low CD34-/PAS+ staining. There were more tumors with high YAP signal among cases with high CD34-/PAS+ staining compared to cases with low CD34-/PAS+ staining (**f**; Chi-square, p = 0.0896). **g**: Two sample t-test comparing transcript levels of YAP/TAZ downstream targets in tumor samples dichotomized into high YAP or low YAP. **h**: Two sample t-test comparing transcript levels of CDC20, CCND1 and ANG2 in tumor samples dichotomized into high CD34-/PAS+ or low CD34-/PAS+.

Supplementary Fig.8

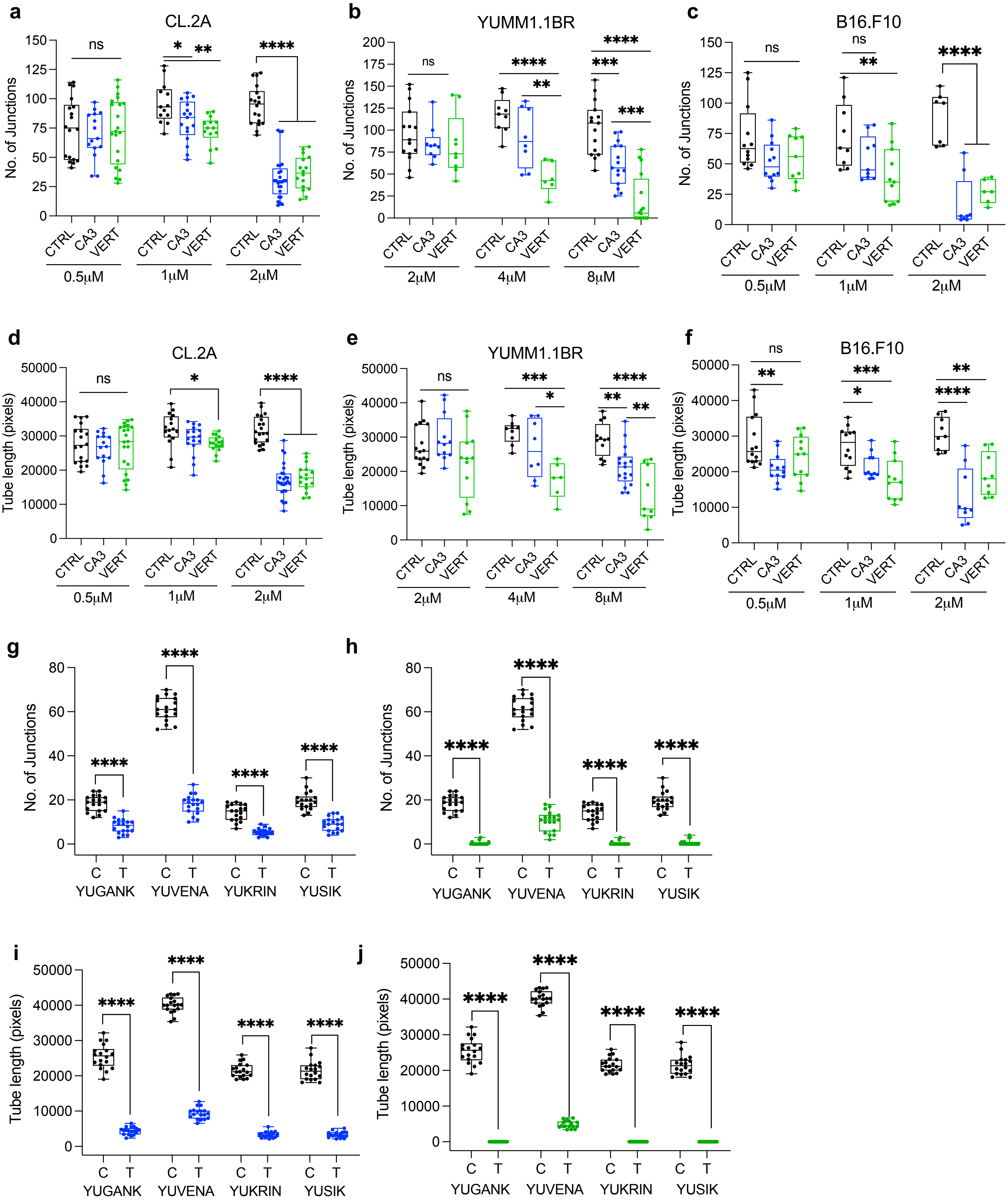

Supplementary Fig. 8

The effect of YAP/TAZ inhibitors on VM junctions and tube length in patient derived and mouse melanoma brain metastasis cultures. Cl.2A (**a**), YUMM1.1Br (**b**), and B16.F10 (**c**) cells were treated with increasing doses of CA3 (blue) and VP (green) for 6 hours at which point VM formation was assessed and number of junctions (**a-c**) and meshes (**d-f**) were quantified. **g-j**: YUGANK, YUVENA, YUKRIN and YUSIK were treated with 0.5μM CA3 (blue) or 0.5μM VP (green) for 6 hours at which point VM formation was assessed, and number of junctions (**g-h**) and tube length (**i-j**) was quantified. All data are expressed as mean ± SD of at least three biological replicates and statistical significance is determined using an unpaired Student's T-test at each time point. ****p<0.0001, ****p<0.001, **p<0.01, *p<0.05.*

Supplementary Fig. 9

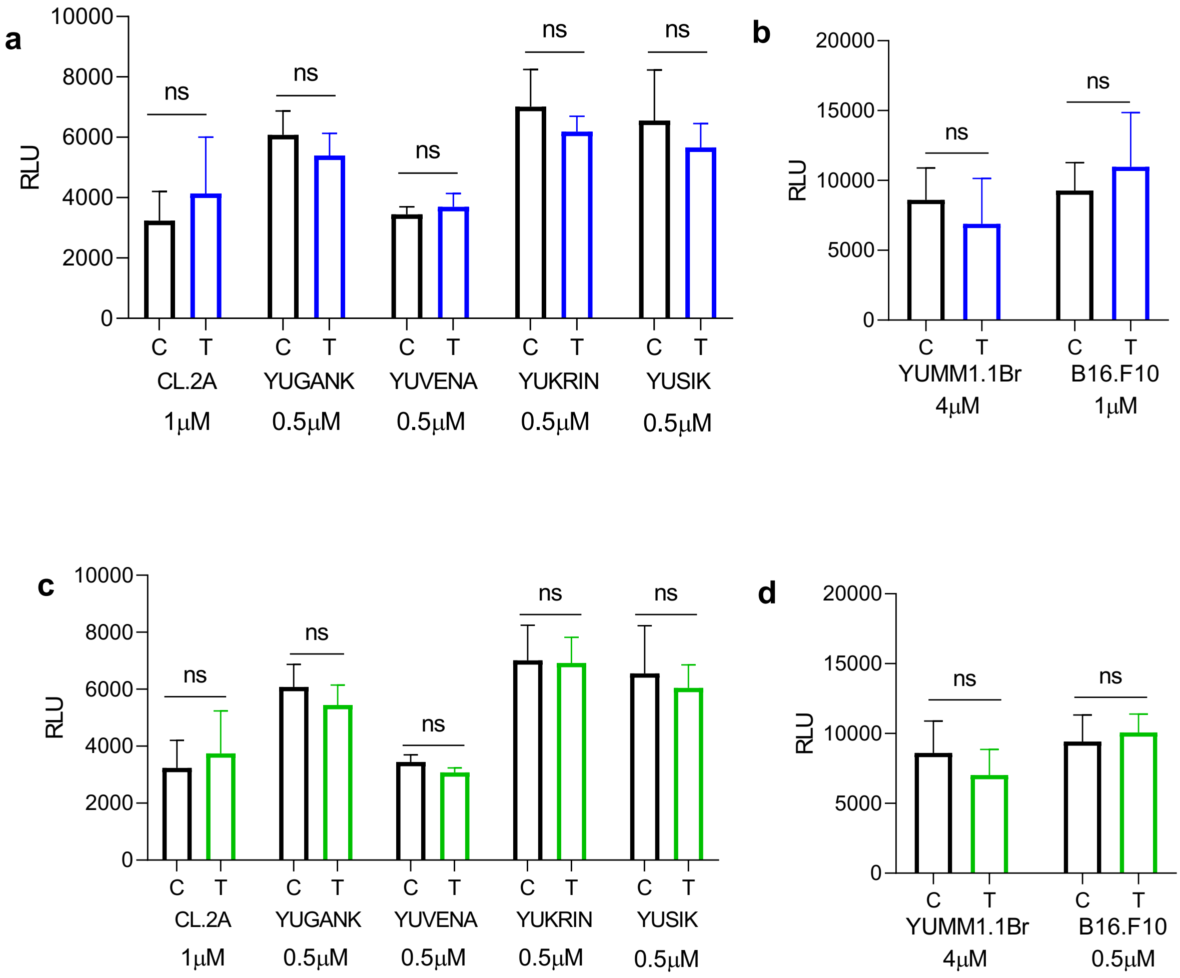

Supplementary Fig. 9

Cell viability at 6-hours after drug treatment. Cell viability after treatment with CA3 (**a-b**) or Verteporfin (**c-d**) at specific doses which inhibit VM in each cell line. Raw relative luminescent units (RLU) values are presented from the DMSO treatment groups (black) and the drug treatment group (blue or green). All data are expressed as mean ± SD of at least three biological replicates and statistical significance is determined using an unpaired Student's T-test at each time point. NS: no significance.

Supplementary Fig. 10

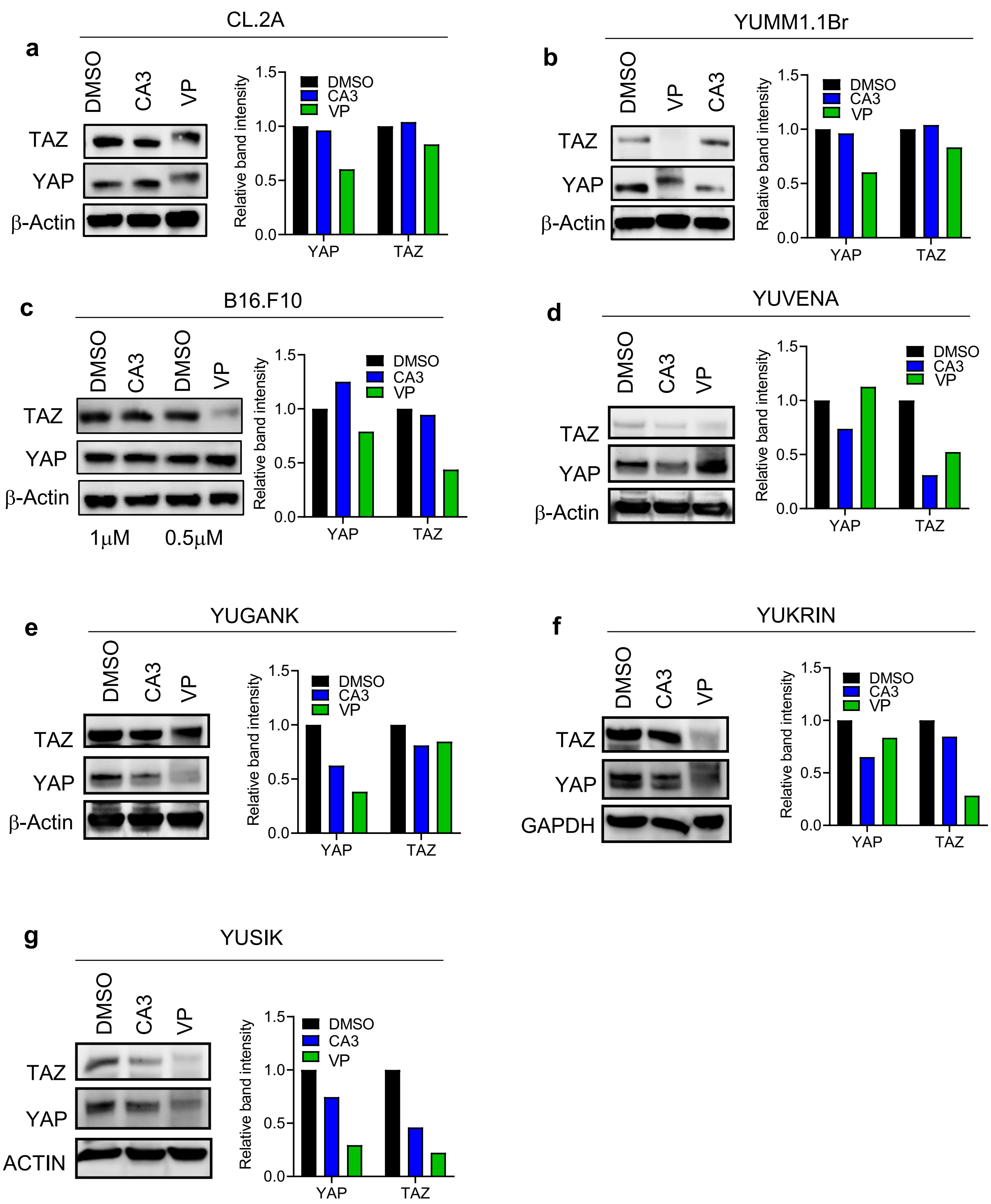

Supplementary Fig.10

The effect of CA3 and Verteporfin on YAP/TAZ signaling. **a:** Western blot (left) and quantification (right) of protein expression in Cl.2A after 1μM DMSO, CA3, or verteporfin treatment in serum starved media for 6 hours. **b:** Western blot (left) and quantification (right) of protein expression in YUMM1.1Br after 4μM DMSO, CA3, or verteporfin treatment in full media for 6 hours. **c:** Western blot (left) and quantification (right) of protein expression in B16.F10 after 1μM treatment of DMSO or CA3 or with 0.5 μM DMSO or verteporfin in serum starved media for 6 hours. Western blot (left) and quantification (right) of protein expression in YUVENA (**d**), YUGANK (**e**), YUKRIN (**f**) and YUSIK (**g**) after treatment with 0.5 μM DMSO, CA3, or verteporfin in serum starved media for 6 hours. For all quantifications, treatment groups were normalized to DMSO. Total-YAP and total-TAZ levels were determined by dividing by the loading control. Western blots are representative of three independent experiments and quantifications are for the western blot shown.

Supplementary Fig. 11

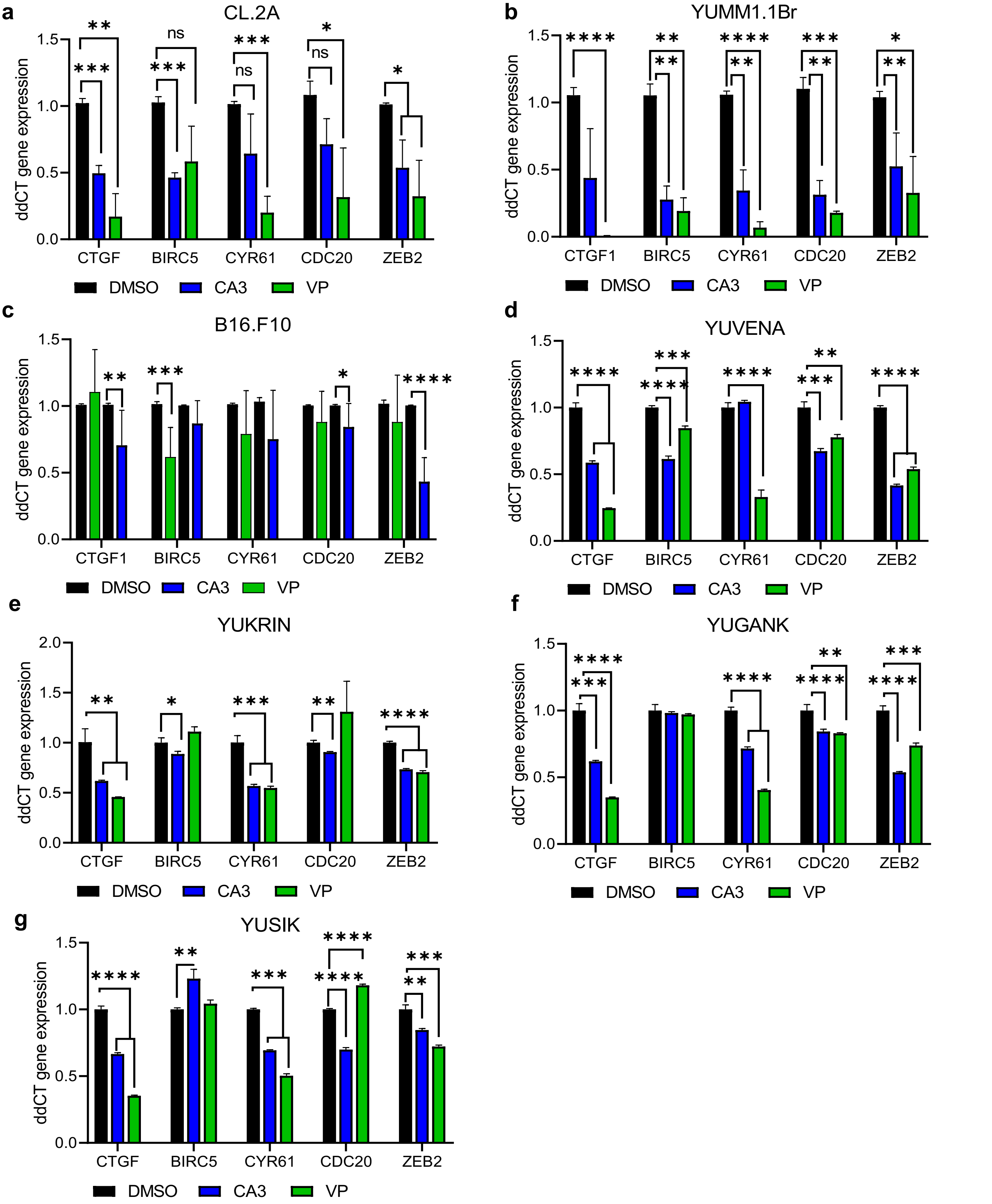

Supplementary Fig.11

The effect of CA3 and Verteporfin on YAP/TAZ target genes **a**: Gene expression in Cl.2A after 1μM DMSO, CA3, or verteporfin treatment in serum starved media for 6 hours. **b**: Gene expression in YUMM1.1Br after 4μM DMSO, CA3, or verteporfin treatment in full media for 6 hours. **c**: Gene expression in B16.F10 after 1μM treatment of DMSO or CA3 or with 0.5 mM DMSO or verteporfin in serum starved media for 6-hours. Gene expression in YUVENA (**d**), YUGANK (**e**), YUKRIN (**f**) and YUSIK (**g**) after treatment with 0.5 μM DMSO, CA3, or verteporfin in serum starved media for 6 hours. Treatment groups were normalized to DMSO. All data are expressed as mean ± SD of at least two biological replicates and statistical significance is determined using an unpaired Student's T-test. Only significance is shown. *$*p<0.05$, $**p<0.01$, $***p<0.001$, $****p<0.0001$.*

Supplementary Fig. 12

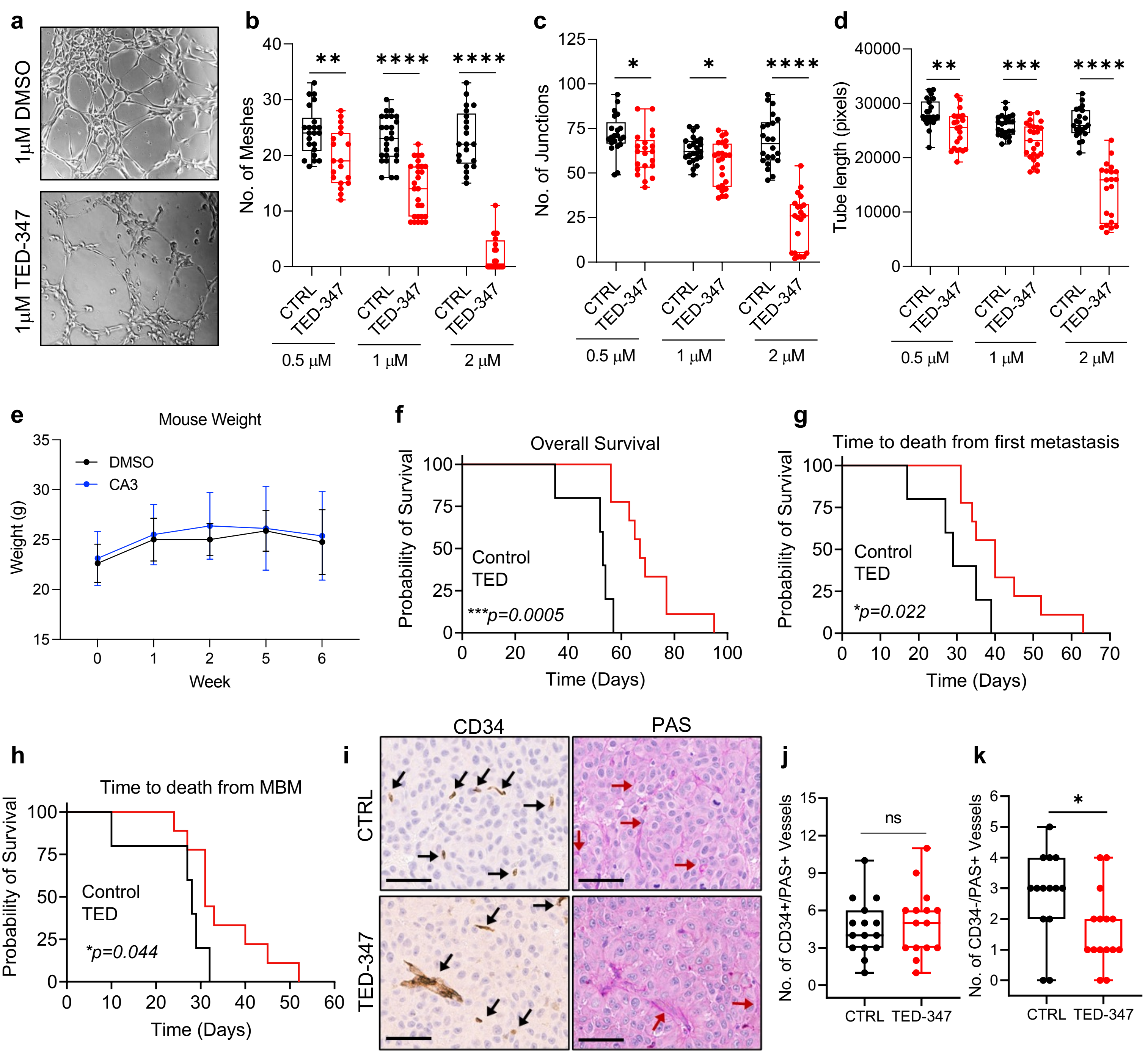

Supplementary Fig. 12
The effect of TED-347 in vitro, CA3 mouse weight, and TED treatment in vivo. **a**: Representative images of VM formation in Cl.2A cells 6 hours after DMSO or 1μM TED-347 treatment at 10x magnification. Quantification of the number of meshes (**b**), number of junctions (**c**), and the total tube length (**d**) at three doses. All data are expressed as mean ± SD of three biological replicates and statistical significance is determined using an unpaired Student's T-test. **e**: Weight of mice treated with CA3 **f-h**: Kaplan-Meier curves for mice receiving vehicle (n=5) or 20mg/kg TED-347 treatment (n=9) in a left ventricle injection murine model of brain metastasis. KM curves demonstrating the correlation between TED-347 treatment and overall survival (**f**), the time to death from first metastasis diagnosis (**g**), and from brain metastasis diagnosis (**h**). **i:** Representative images of immunohistochemical staining for CD34 and PAS in Cl.2A brain tumors developed after left-ventricle injection in nude mice treated as controls, or with 20mg/kg TED-347. Black arrows point to BV, red arrows point to VM structures. Scale bars represent 50μM. Quantification of BV (**j**; CD34+/PAS+) and VM (**k**; CD34-/PAS+) 15 areas from at least 2 tumors from each treatment. Students t-test was used to assess significance between vascular density in control and TED-347 treated mice. *p<0.05

Supplementary Fig. 13

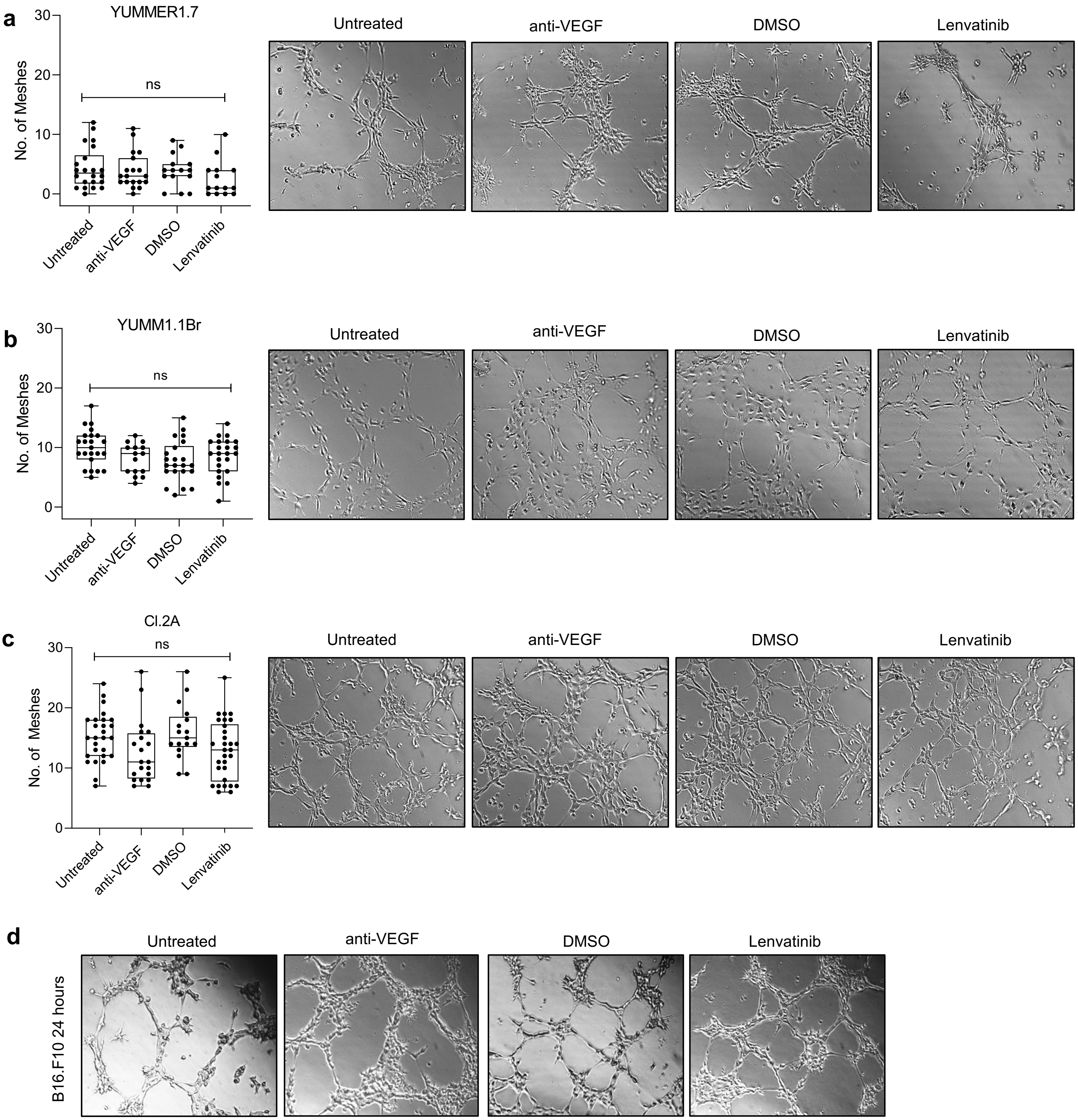

Supplementary Fig.13

Angiogenesis inhibitors do not reduce VM in vitro. The effect of lenvatinib and anti-VEGF on VM formation in the mouse melanoma cell lines YUMMER1.7 (**a**), YUMM1.1BR (**b**) after 4-6 hours of treatment. (**c**) The effect of lenvatinib and anti-VEGF (bevacizumab) in the human melanoma cell line Cl.2A after 4-6 hours of treatment. VM formation was assessed by mesh quantification. (**d**) B16.F10 mouse melanoma cells after 24-hour treatment with lenvatinib and anti-VEGF. For all cell lines, anti-VEGF concentration was 1mg/mL and lenvatinib was 10μM.

Supplementary Fig. 14

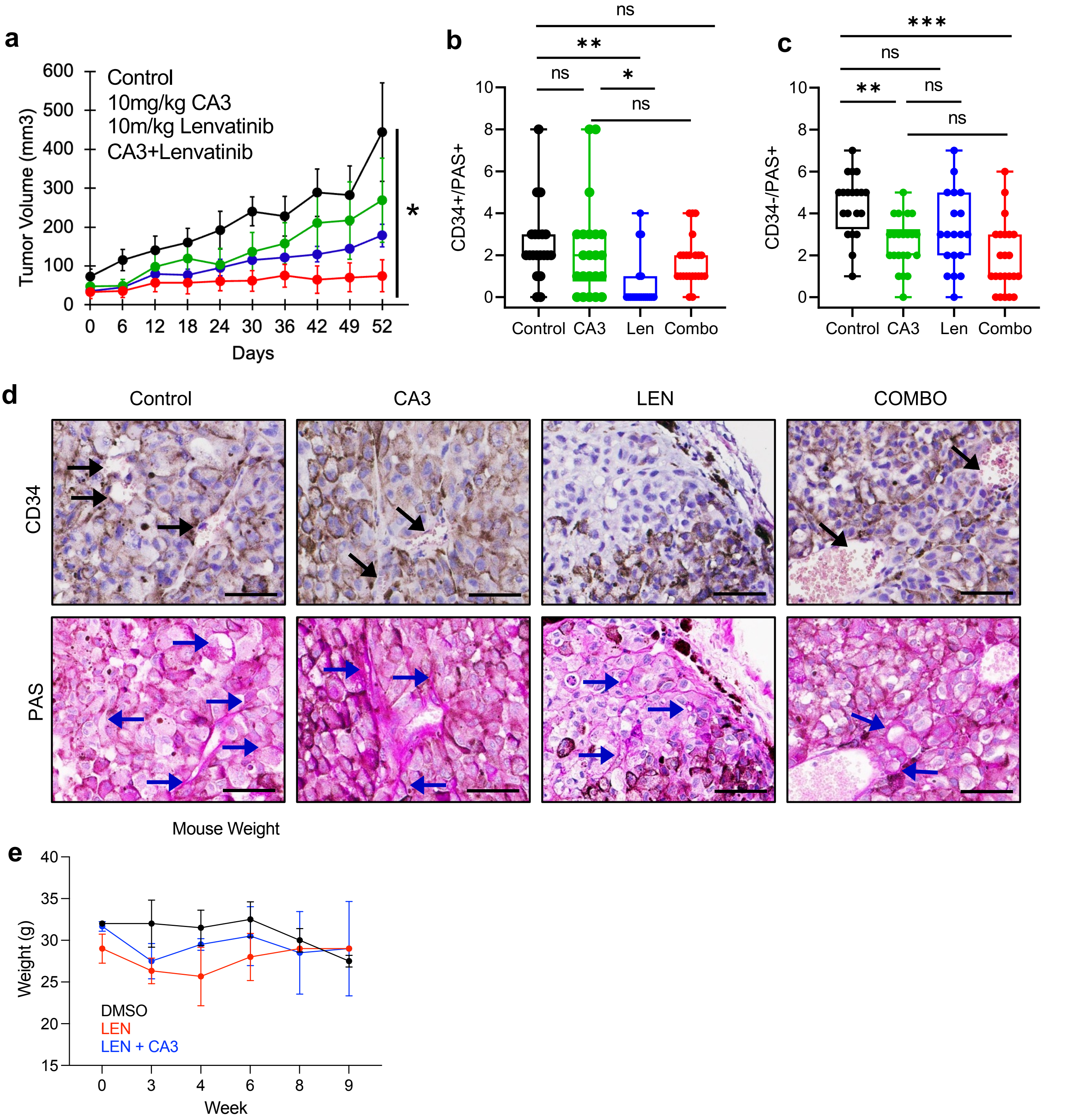

Supplementary Fig.14

Combined anti-angiogenic and YAP directed therapies on anti-tumor responses. **a.** Tumor growth curve of mice with YUSIK subcutaneous tumors at 52 days after tumor implantation (Control n=6, CA3 n=5, Lenvatinib n=5, Combo n=4). CA3 and lenvatinib alone had no benefit compared to control (p=0.346 and p=0.113, respectively). Combination treatment significantly reduces tumor growth compared to control (p=0.042). Lenvatinib versus combination treatment trends toward significance (p=0.085). Statistical significance is determined using t-test compared to control ****p<0.0001, ***p<0.001, *p<0.05.* Quantification of the number of BV (**b**) and VM (**c**) in at least 6 areas from three tumors of each treatment group. (**d**) Representative images of immunohistochemical staining for CD3 and PAS in YUSIK subcutaneous tumors. Black arrows point to BV's (CD34+/PAS+ structures) and blue arrows point to VM structures (CD34-/PAS+). Scale bars represent 50µM. **e.** Average mouse weight overtime in each treatment cohort. Statistical significance is determined using a one-way ANOVA with multiple comparisons ****p<0.0001, ***p<0.001, *p<0.05.*
